# Argonaute binding within 3′-untranslated regions poorly predicts gene repression

**DOI:** 10.1093/nar/gkaa478

**Published:** 2020-06-05

**Authors:** Yongjun Chu, Audrius Kilikevicius, Jing Liu, Krystal C Johnson, Shinnichi Yokota, David R Corey

**Affiliations:** UT Southwestern Medical Center, Departments of Pharmacology and Biochemistry, Dallas, TX 75205, USA; UT Southwestern Medical Center, Departments of Pharmacology and Biochemistry, Dallas, TX 75205, USA; UT Southwestern Medical Center, Departments of Pharmacology and Biochemistry, Dallas, TX 75205, USA; UT Southwestern Medical Center, Departments of Pharmacology and Biochemistry, Dallas, TX 75205, USA; UT Southwestern Medical Center, Departments of Pharmacology and Biochemistry, Dallas, TX 75205, USA; UT Southwestern Medical Center, Departments of Pharmacology and Biochemistry, Dallas, TX 75205, USA

## Abstract

Despite two decades of study, the full scope of RNAi in mammalian cells has remained obscure. Here we combine: (i) Knockout of argonaute (*AGO*) variants; (ii) RNA sequencing analysis of gene expression changes and (iii) Enhanced Crosslinking Immunoprecipitation Sequencing (eCLIP-seq) using anti-AGO2 antibody to identify potential microRNA (miRNA) binding sites. We find that knocking out *AGO1*, *AGO2* and *AGO3* together are necessary to achieve full impact on steady state levels of mRNA. eCLIP-seq located AGO2 protein associations within 3′-untranslated regions. The standard mechanism of miRNA action would suggest that these associations should repress gene expression. Contrary to this expectation, associations between AGO and RNA are poorly correlated with gene repression in wild-type versus knockout cells. Many clusters are associated with increased steady state levels of mRNA in wild-type versus knock out cells, including the strongest cluster within the *MYC* 3′-UTR. Our results suggest that assumptions about miRNA action should be re-examined.

## INTRODUCTION

RNA interference (RNAi) is a powerful mechanism for controlling steady state levels of mRNA in mammalian cells (1,2) and is now achieving success in the clinic (3,4). Chromosomally-encoded hairpin RNAs are expressed and are processed into microRNAs (miRNAs) (5). These miRNAs are loaded into argonaute protein (AGO) to form a ribonucleoprotein in which the miRNA directs the complex to bind complementary RNA sequences. AGO increases the efficiency of binding and allows association with GW182 and other proteins to enhance function (6,7). miRNAs are thought to require only partial complementarity to target sequences within 3′-untranslated regions (3′-UTRs), with a match at bases 2–8 being most important for recognition (8,9).

There are four AGO proteins in human cells, AGO1–4 (6). AGO2 is the best studied and is known as the catalytic engine for RNAi because it possesses an enzyme active site capable of cleaving target RNA transcripts (10,11). This ability to cleave mRNA is critical for applying fully complementary synthetic RNAs to gene silencing and contributes to the robustness of RNAi as a method for controlling gene expression in the laboratory and the clinic. The roles of AGO1 and AGO3 are less known. Reports have suggested that miRNAs are randomly loaded into AGO1, AGO2, and AGO3 according to their individual cellular abundance (12–14). AGO4 had been observed to make a negligible contribution to RNAi in the cell lines tested (12).

The current mechanistic model describing the action of miRNAs suggests that RNAi proteins promote seed sequence binding of miRNAs within 3′-UTRs leading to mRNA degradation and reduction of mRNA levels (15). The complex between AGO protein and miRNA nucleates the formation of a larger protein complex that recruits the CCR4-NOT complex that promotes mRNA decay (16). These miRNA-directed interactions would be expected to lead to decreased steady state levels of target mRNA that can be detected by RNA sequencing (RNAseq).

Since 2000, a literature search of the term miRNA reveals over 90 000 citations with over 10 000 new citations appearing every year. 42 000 papers appear on a PubMed search of ‘cancer’ and ‘miRNA’. These numbers suggest that the science of miRNA action is well-established. The assumption that miRNAs should have widespread roles in regulating cell biology has been based on several factors: (i) efficient RNAi gene regulation in *C. elegans* and other model organisms (17–19); (ii) the simplicity of base-pairing rules that encourage hypotheses connecting a miRNA seed sequence to a potential target gene of interest and (iii) the known robustness of fully complementary synthetic RNAs as gene silencing agents in mammalian cells (20).

Closer examination, however, reveals that many papers lack the minimum controls and experimentation necessary to make convincing conclusions linking complementary recognition by a miRNA to a functional effect on gene expression (21). One possible reason for the proliferation of these studies claiming diverse and often conflicting regulatory roles for miRNAs is an inadequate understanding of the quantitative and biochemical foundations for RNAi and miRNAs inside human cells.

The standard expectation for the action of miRNAs suggests that knocking out *AGO* gene expression should reverse the action of miRNAs and increase the expression of genes with significant engagement between 3′-UTRs and AGO protein. Here we test this assumption by combining enhanced crosslinking immunoprecipitation (eCLIP) identifying the locations for AGO binding within 3′-UTRs with analysis of the impact of *AGO1*, *AGO2*, *AGO1/2* and *AGO1/2/3* gene knockouts.

Contrary to long-standing expectations, we observed that AGO binding within 3′-UTRs did not show a useful correlation with increased steady state levels of mRNA when *AGO* genes are knocked out. Instead, most genes with AGO:3′-UTR associations either show no significant changed or decreased expression. Knocking out AGO1, AGO2 and AGO3 protein expression was necessary to achieve full effects, regardless of the direction of expression change. While the magnitude of effects became much greater as more *AGO* genes are knocked out, the direction of change for individual genes tended to remain the same.

The lack of correlation between AGO:3′-UTR association and gene repression suggests that a simple connection between miRNA engagement and gene repression cannot be assumed. Important questions about the role of AGO:miRNA function remain unanswered and addressing these questions may reveal unanticipated pathways for RNA regulation by AGO and other RNAi proteins.

## MATERIALS AND METHODS

### Cell lines

The HCT116 cell line (Horizon Discovery) originated from the American Type Culture Collection (ATCC) and then licensed and supplied to the European Collection of Authenticated Cell Cultures (ECACC). ATCC authenticated this HCT116 cell line using Short Tandem Repeat (STR) analysis as described in 2012 ANSI Standard (ASN-0002) Authentication of Human Cell Lines: Standardization of STR profiling by the ATCC Standards Development Organization (SDO) (22). The ATCC STR analysis compared seventeen short tandem repeat loci plus the gender-determining locus, Amelogenin, to verify the HCT116 cell line (ATCC CCL 247). ECACC performed an additional STR analysis of seventeen loci on the cells received from ATCC, and the verified HCT116 cells (ECACC 91091005) were supplied to Horizon Discovery.

**Figure 1.**
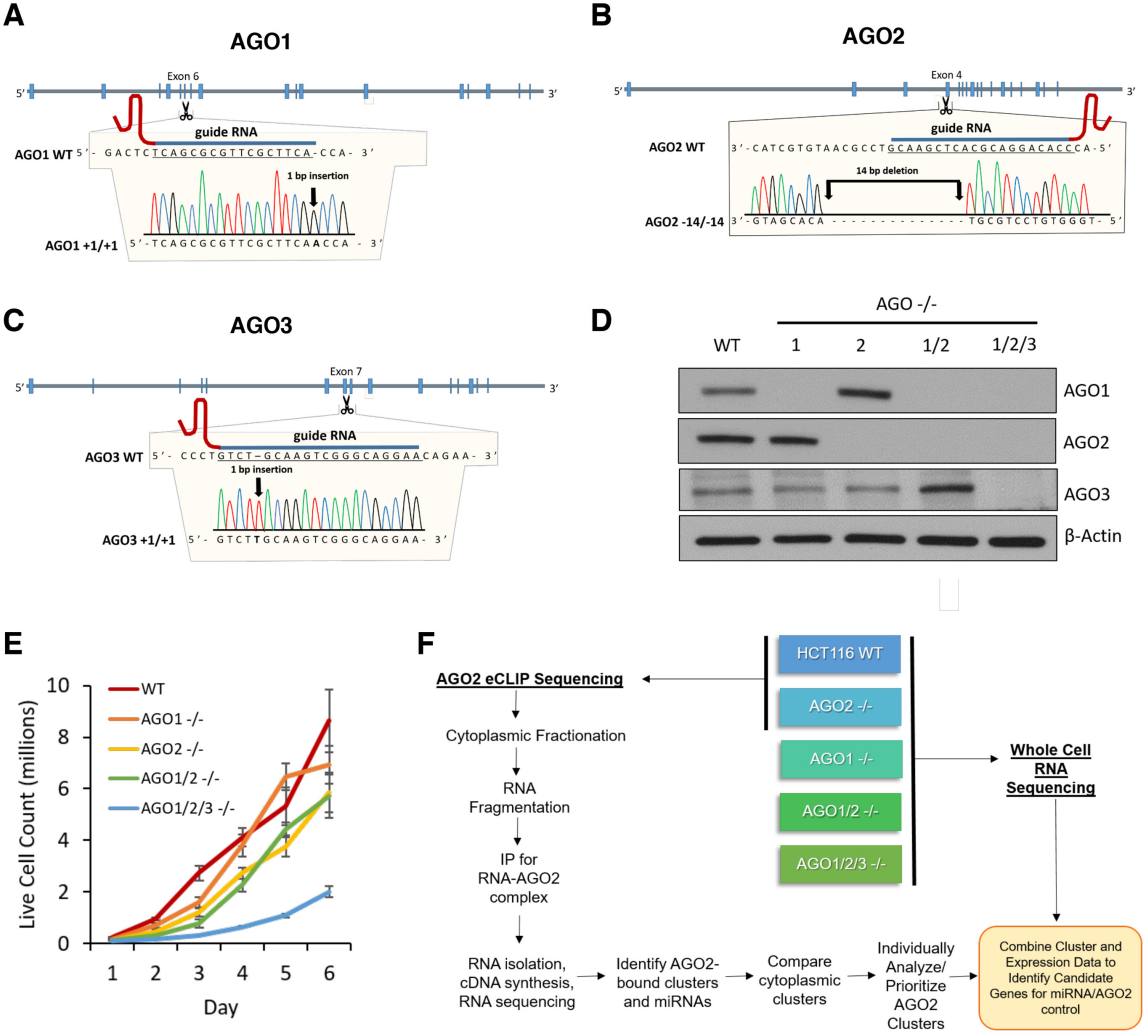
Characterization of CRISPR-derived knockout cell lines. (**A–C**) Location of guide RNAs and mutations knocking out *AGO1*, *AGO2* and *AGO3* expression. (**D**) Western blot validating the *AGO1*, *AGO2*, *AGO1/2* and *AGO1/2/3* knockout cell lines (n = 3). (**E**) Effect of knockouts on growth rates. WT, *AGO1*, *AGO2*, *AGO1/2* and *AGO1/2/3* knockout cell lines were seeded at the same density, harvested, and analyzed for live cell count (n = 3). A Two-Way ANOVA with Bonferroni post-test analysis show that the *AGO2−/−* (*P* value 0.0066), *AGO1/2−/−* (*P* value 0.0045), and AGO1/2/3*−/−* (*P* value 0.0002) growth rates are significantly different from WT. (**F**) Scheme showing knockout of AGO variants, RNAseq analysis of gene expression changes relative to wild-type cells; and eCLIP-sequencing with anti-AGO2 antibody to identify potential miRNA–AGO2 binding sites. See also Supplemental Figure S1.

For eCLIP, we used HCT116:AGO2 knockout cells obtained from Joshua T. Mendell (UT Southwestern) (23). The *AGO1*, *AGO2*, *AGO1/2* and *AGO1/2/3* knockout cell lines used for RNAseq were prepared using GenCRISPR™ gene editing technology and services (GenScript) and verified HCT116 cells (Horizon Discovery). The AGO2 knockout cell line was independently generated to ensure that all cell lines used for RNAseq were derived from similar genetic backgrounds. gRNA sequence, gRNA target locations, and DNA sequencing results are shown in Figure 1. All HCT116 and HCT116-derived cells were cultured in McCoy's 5A Medium (Sigma-Aldrich) supplemented with final 10% FBS at 37°C in 5% CO_2_. For the cell growth assay, the cells were seeded at a density of 50 000 cells/mL, disassociated with 1× trypsin, and counted using trypan blue staining (TC20 Automated Cell Counter, Bio-Rad).

### Preparation of cytoplasmic extract

Cytoplasm isolation was similar as previously described (24,25), with modifications for HCT116 and HCT116-derived cells. Cells at ∼95% confluence were lysed in hypotonic lysis buffer (HLB) (10 mM Tris–HCl, pH 7.4, 10 mM NaCl, 3 mM MgCl_2_, 2.5% NP-40, 0.5 mM DTT, 1× protease inhibitor (Roche, Complete) and 50 U/ml ribonuclease inhibitors (RNasin^®^ Plus, Promega) and supernatant collected as cytoplasmic fraction. Western blots to determine the purity of fractions and RNAi factors distribution were performed using western-blot analysis as before (25).

### Western-blot analysis

Total protein lysate was prepared re-suspending cells in lysis buffer (50 mM Tris–HCl, pH 7.0, 120 mM NaCl, 0.5% NP-40, 1 mM EDTA, 1 mM DTT, 1× protease inhibitor (Roche, cOmplete). Protein were separated on 4–20% gradient Mini-PROTEAN^®^ TGXTM precast gels (Bio-Rad). After gel electrophoresis, proteins were wet transferred to nitrocellulose membrane (0.45 μm, GE Healthcare Life Sciences) at 100 V for 75 min. Membranes were blocked for 1 h at room temperature with 5% milk in 1× PBS containing 0.05% TWEEN-20. Blocked membranes were incubated with the primary antibodies in blocking buffer at 4°C on rocking platform overnight: using anti-AGO1, 1:2000 (5053, Cell Signaling), anti-AGO2, 1:1500 (015-22031, Fujifilm WAKO), anti-AGO3, 1:500 (39787, Active Motif), anti-Calnexin, 1:1000 (2433, Cell Signaling), anti-LaminA/C, 1:1500 (ab8984, Abcam), anti-GAPDH, 1:800 (ab9484, Abcam) and anti-ATP Synthase subunit β, 1:1000 (A-21351, Invitrogen), anti-β-actin, 1:15000 (A5441, Sigma-Aldrich), anti-CEMIP (KIAA1199), 1:1000 (ab98947, Abcam), anti-GALNT3, 1:1000 (AF7174, R&D systems), anti-c-MYC, 1:500 (13-2500, Invitrogen) antibodies. After primary antibody incubation, membranes were washed 3 × 10 min at room temperature with 1× PBS+0.05% Tween-20 (PBST 0.05%) and then incubated for 1 h at room temperature with respective secondary antibodies in blocking buffer. Membranes were washed again 4 × 10 min in PBST 0.05%. Washed membranes were soaked with HRP substrate according to manufacturer's recommendations (SuperSignal™ West Pico Chemiluminescent substrate, Thermo Scientific) and exposed to films. The films were scanned and bands were quantified using ImageJ software.

### RT-qPCR

Total RNA was extracted from HCT116 wild-type or knockout cells and treated with DNase I (Worthington Biochemical) at 25°C for 20 min, 75°C for 10 min. Reverse transcription was performed using high-capacity reverse transcription kit (Applied Biosystems) per the manufacturer's protocol. 2.0 μg of total RNA was used per 20 μl of reaction mixture. PCR was performed on a 7500 real-time PCR system (Applied Biosystems) using iTaq SYBR Green Supermix (BioRad). PCR reactions were done in triplicates at 55°C 2 min, 95°C 3 min and 95°C 30 s, 60°C 30 s for 40 cycles in an optical 96-well plate. For each gene, two different sets of primer were used to check mRNA level (Supplementary Table S1). Data were normalized relative to measured hypoxanthine phosphoribosyltransferase 1 (HPRT1) and Small Nuclear Ribonucleoprotein U5 Subunit 200 (snRNP200) genes level.

### Determining miRNA copy number

Cells were disassociated and counted with Trypan blue as described above. Total RNA was extracted from HCT116 wild-type cells and treated with DNase I (Worthington Biochemical) at 25°C for 20 min, 75°C for 10 min. Synthetic oligonucleotide miRNA mimics were synthesized by IDT and diluted to 100 μM in nuclease free water. The synthetic oligonucleotide miRNA mimics were used for a 10-fold serial dilution in nuclease free water supplemented with RNase Inhibitor in 0.5 ml DNA LoBind Tubes (Eppendorf). Reverse transcription was performed using microRNA high-capacity reverse transcription kit (Applied Biosystems) per the manufacturer's protocol. PCR was performed on a 7500 real-time PCR system (Applied Biosystems) using TaqMan Universal PCR Master Mix No AmpErase UNG (Applied Biosystems) with the primers and probes included in the Taqman microRNA Assay kits (hsa-miR-29a-3p: Assay ID: 002112; hsa-miR-125b-5p: Assay ID: 000449; hsa-let-7a-5p: Assay ID: 000377; hsa-let-7f-5p: Assay ID: 000382; hsa-miR-21-5p: Assay ID: 000397; hsa-miR-27a-3p: Assay ID: 000408). PCR reactions were done in triplicates at 55°C 2 min, 95°C 3 min and 95°C 30 s, 60°C 30 s for 40 cycles in an optical 96-well plate. The CT values were plotted against the titrated synthetic oligonucleotide miRNA concentration (log_10_-scale), and analyzed with linear regression to calculate the copy number per cell in the HCT116 wild-type cells.

### RNAseq analysis of steady state mRNA levels

WT HCT116, *AGO1*, *AGO2*, *AGO1/2* and *AGO1/2/3* knock out cells were used for RNAseq. Three biological replicated samples were sequenced. Approximately 3.0 × 10^6^ cells were seeded in a 15-cm large dish. Cells were harvested 48 h later and RNA was extracted using the RNeasy Mini Kit (Qiagen) with an on-column DNase digestion. Sequencing libraries were generated using the TruSeq Stranded Total RNA with Ribo-Zero Human/ Mouse/Rat Low-throughput (LT) kit (Illumina) and run on a NextSeq 500 for paired-end sequencing using the NextSeq 500/550 High Output v2 Kit, 150 cycles (Illumina).

Quality assessment of the RNAseq data was done using NGS-QC-Toolkit43 with default settings. Quality-filtered reads generated by the tool were then aligned to the human reference genome hg38 and transcriptome Gencode v75 using the STAR (v 2.5.2b) using default settings. Read counts obtained from STAR were used as input for Salmon (v 1.0.0) and Deseq2 for gene differential analysis of steady state RNA levels. Genes with adjusted *P* ≤ 0.05 were regarded as differentially expressed for comparisons of each sample group.

### Preparation of RNAseq libraries

Control and *AGO2*−/− HCT116 cells (obtained from Dr. Joshua Mendel, UT Southwestern) were seeded in 15 cm dishes with 12 dishes per cell line at 3.0 × 10^6^ cells per dish. Cells were cultured for 48 h and subsequently UV crosslinked at 300 mJ/cm^2^. Cytoplasmic fraction was collected as described above. eCLIP was performed using the frozen samples as previously described (26), using anti-AGO2 antibody for IPs (3148, gift from Jay A. Nelson lab). For each cell line, duplicate input and IP samples were prepared and sequenced. The RiL19 RNA adapter (Supplementary Table S2) was used as the 3′ RNA linker for input samples. RNA adapters RNA_A01, RNA_B06, RNA_C01, RNA_D08, RNA_X1A, RNA_X1B, RNA_X2A, RNA_X2B were used for IP samples (Supplementary Table S2). PAGE purified DNA oligonucleotides were obtained from IDT for the PCR library amplification step (Supplementary Table S2). PCR amplification was performed using between 11 and 16 cycles for all samples. Paired-end sequencing was performed on a NextSeq 500 using the NextSeq 500/550 High Output v2 Kit, 100 cycle (Illumina).

### Mapping deep sequencing reads

Adapters were trimmed from original reads using Cutadapt (v1.9.1) with default settings. Next, the randomer sequence from the rand103Tr3 linker (Supplementary Table S2) was trimmed and recorded. STAR (v2.5.2b) was used to align mate 2 to hg38. Only the uniquely mapped reads were retained. PCR duplicates were then removed using the randomer information using an in-house script. All reads remaining after PCR duplicate removal were regarded as usable reads and used for cluster calling. The usable reads number and reproducibility between biological replicates met highest standards of eCLIP experiments (Supplementary Figure S2C, D).

### eCLIP cluster calling and annotation

eCLIP clusters were identified following a method described by original eCLIP developers (26) with the following modifications. Initial AGO2 binding clusters identified by CLIPper (27) in wild-type HCT116 were filtered to keep only clusters that are statistically significant (*P* < 0.001). For each region, normalization to total usable reads was performed and a fold change between IP and combined samples (input and IP in knockout cell line samples) was calculated. Significant CLIP clusters in each dataset were defined by (i) *P* < 0.05 determined by the Fisher exact test or Yates’ Chi-square test and (ii) log_2_ fold change of normalized reads in the cluster was ≥2 comparing IP to combined (input + IP in knockout cells).

The final CLIP clusters for AGO2 were identified by first identifying significant clusters present in both experimental replicates. A cluster was considered to be present in both replicates if it occurred on the same strand and the replicate clusters overlapped by at least one-third of their total length. Significant clusters from both replicates were then merged to define the final cluster length. Clusters were annotated based on their genomic locations (Gencode v27). If a cluster was assigned to multiple annotations, the annotation was selected using the following priority: CDS exon > 3′ UTR > 5′ UTR > protein-coding gene intron > noncoding RNA exon > noncoding RNA intron > intergenic.

### Estimation of steady state miRNA Levels from eCLIP-seq

Bowtie2 was used to map the miRNA tags to the miRBase (mature miRNAs), allowing up to one mismatch. The miRNA levels are quantified as the number of reads mapped to individual miRNAs normalized by the total number of mapped reads in miRBase. The miRNA levels from different samples were further normalized by quantile normalization in order to control for batch effect.

### Statistical analysis

The dynamic and bar graphs represent mean and standard deviation. The averages among cells we compared using one or two ways analysis of variance followed by Bonferroni and Tukey post-hoc tests (*P* < 0.05). To determine correlation between AGO2 binding cluster significance level and the change of steady state mRNA levels in AGO knockout cells, we first tested data sets for normal distribution (D’Agostino and Pearson omnibus normality test, Kolmogorov–Sminov test). If both correlating data sets could not pass normality test, or were not linear, we calculated Spearman's correlation coefficient.

## RESULTS

### Knockout of AGO1, AGO2 and AGO3

We obtained CRISPR gene knockouts to investigate the role of AGO in controlling steady state levels of mRNA. HCT116 colorectal cancer cells were chosen as a parental line because they are diploid, simplifying the process of obtaining CRISPR-mediated double or triple gene knockouts. HCT116 is a typical cell line in terms of miRNA levels (28) (Supplementary Figure S1).

The *AGO1*, *AGO2*, *AGO1/2* and *AGO1/2/3* knockout cells were verified by DNA sequencing (Figure 1A–C). Western analysis confirmed that the knock out cell lines did not express the targeted proteins (Figure 1D). *AGO4* KO cells were not obtained because we had previously used quantitative mass spectrometry to determine that AGO4 protein was not expressed at detectable levels in wild-type HCT116 cells (29).

We evaluated the growth rates of the knockout cell lines relative to wild-type cells (Figure 1E). The growth rates of *AGO1* knockout and wild-type cells were similar. *AGO2* and *AGO1/2* knockout cells grew more slowly than wild-type cells. The *AGO1/2/3* triple knock out cells grew at the slowest rate, consistent with the conclusion that knocking out all three primary *AGO* variants leads to a greater physiologic effect than could be achieved in the single or double *AGO* knockout cells.

Enhanced crosslinking immunoprecipitation (eCLIP) RNAseq is a version of CLIP designed to increase signal to noise and improve confidence in the detection of potential sites of protein:RNA association (26). To determine sites of AGO binding we used an anti-AGO2 antibody. We focused on AGO2 because it is the best-studied AGO variant and because the anti-AGO2 antibody was well-characterized as efficient for pull-down experiments (30,31). We used RNAseq to measure whole cell RNA levels in wild-type and knockout HCT116 cells for all *AGO* knockout cell lines. The correlation of AGO-binding data and gene expression data allowed us to classify the effect of *AGO* knockouts on steady state mRNA levels associated with genes possessing significant clusters of eCLIP RNAseq reads due to engagement of AGO2 (Figure 1F) with data presented below.

### Distribution of AGO2 binding sites

For eCLIP, we isolated RNA from the cytoplasmic fraction of HCT116 cells (Supplementary Figure S2). Parallel experiments were performed on HCT116 cells with *AGO2* gene expression knocked out to subtract background signal. The significance of clusters was initially evaluated using the CLIper peak calling program (27), and then re-calculated using the Fisher exact test or Yates’ Chi-square test which considers the combined signals from Input and AGO2-eCLIP with *AGO2* KO cells. A *P*-value <0.05 and an enrichment fold over 4 for wild-type versus *AGO2* knockout cells were applied as a threshold for a peak to be included in our detailed analysis. All clusters were individually curated and visually examined to gain insights into biological significance and potential mechanisms of action.

**Figure 2.**
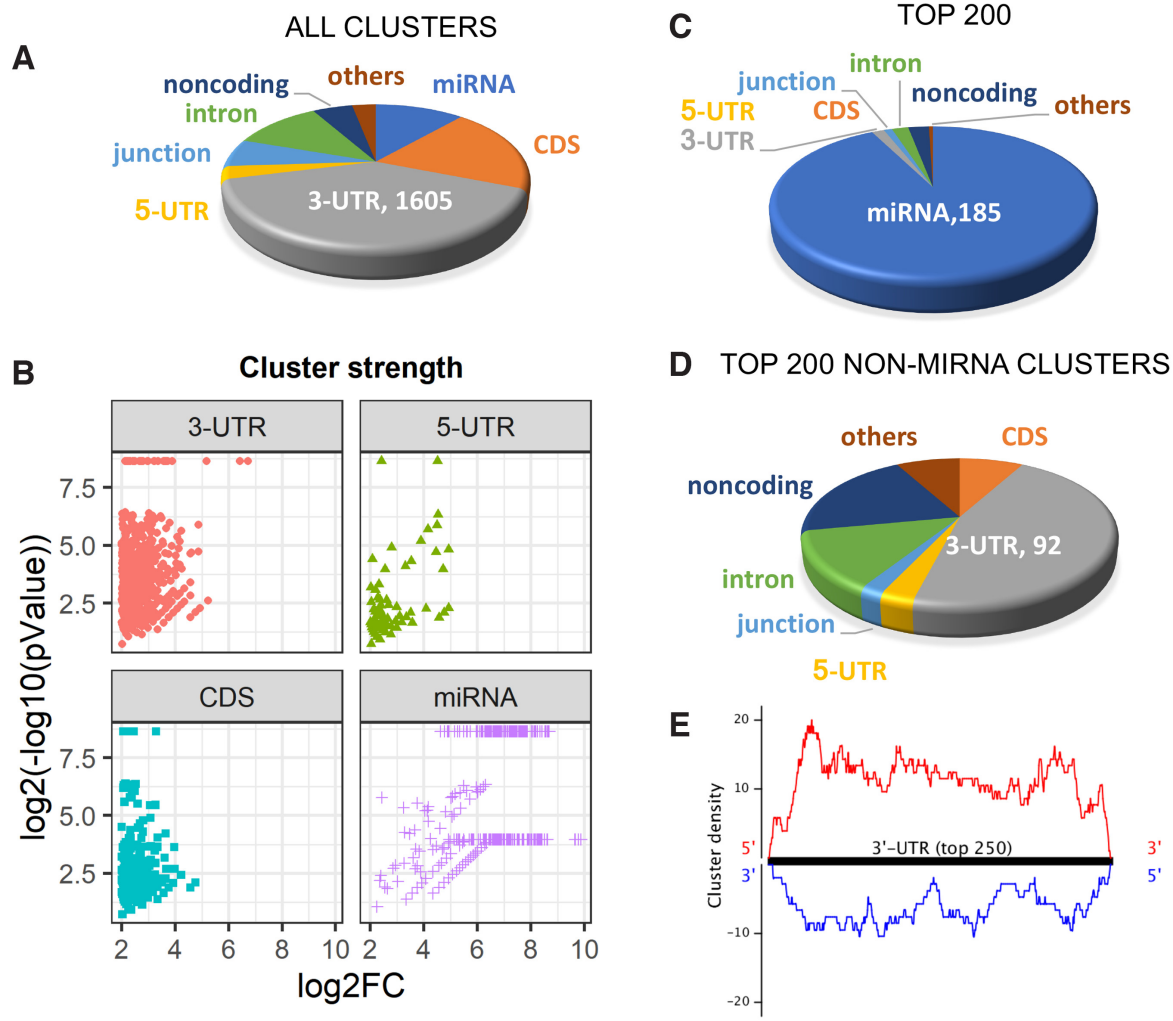
Localization AGO2 engagement with cytoplasmic RNA disclosed by eCLIP. (**A**) Relative distribution of all AGO-binding clusters within cytoplasmic RNA. (**B**) Strength of clusters within different transcript regions. The Y axis represents log_2_ values for cluster significance. The X axis is log_2_ fold change number relative to input and AGO2 knockout samples. (**C**, **D**). Relative distribution of of top clusters, ranked by significance for the top 200 clusters and the top 200 clusters that that are not directly associated with miRNAs. (**E**) Localization of the strongest 250 clusters within 3′-UTRs. See also Supplemental Figure S2.

Consistent with previous results (32–35) the largest category of AGO2 binding sites detected by eCLIP were within the 3′-UTR of genes (Figure 2A). In many cases, genes contained multiple binding sites: 1077 genes possessed at least one significant cluster of sequencing reads within their 3′-UTRs.

While many clusters were within 3′-UTRs, most high-ranking clusters were due to AGO2 binding with miRNAs including 90% of the top two hundred clusters (Figure 2B and C). Strong association with miRNAs may be because AGO2 binds directly to miRNA and because miRNAs tend to be much more highly expressed than mRNAs (36). Of the top 200 clusters that were not miRNAs, 50% were within the 3′-UTR (Figure 2D). Examination of the top 250 clusters within the 3′-UTR revealed that AGO binding sites are distributed evenly throughout the region (Figure 2E).

### miRNA levels in HCT116 cells

**Figure 3.**
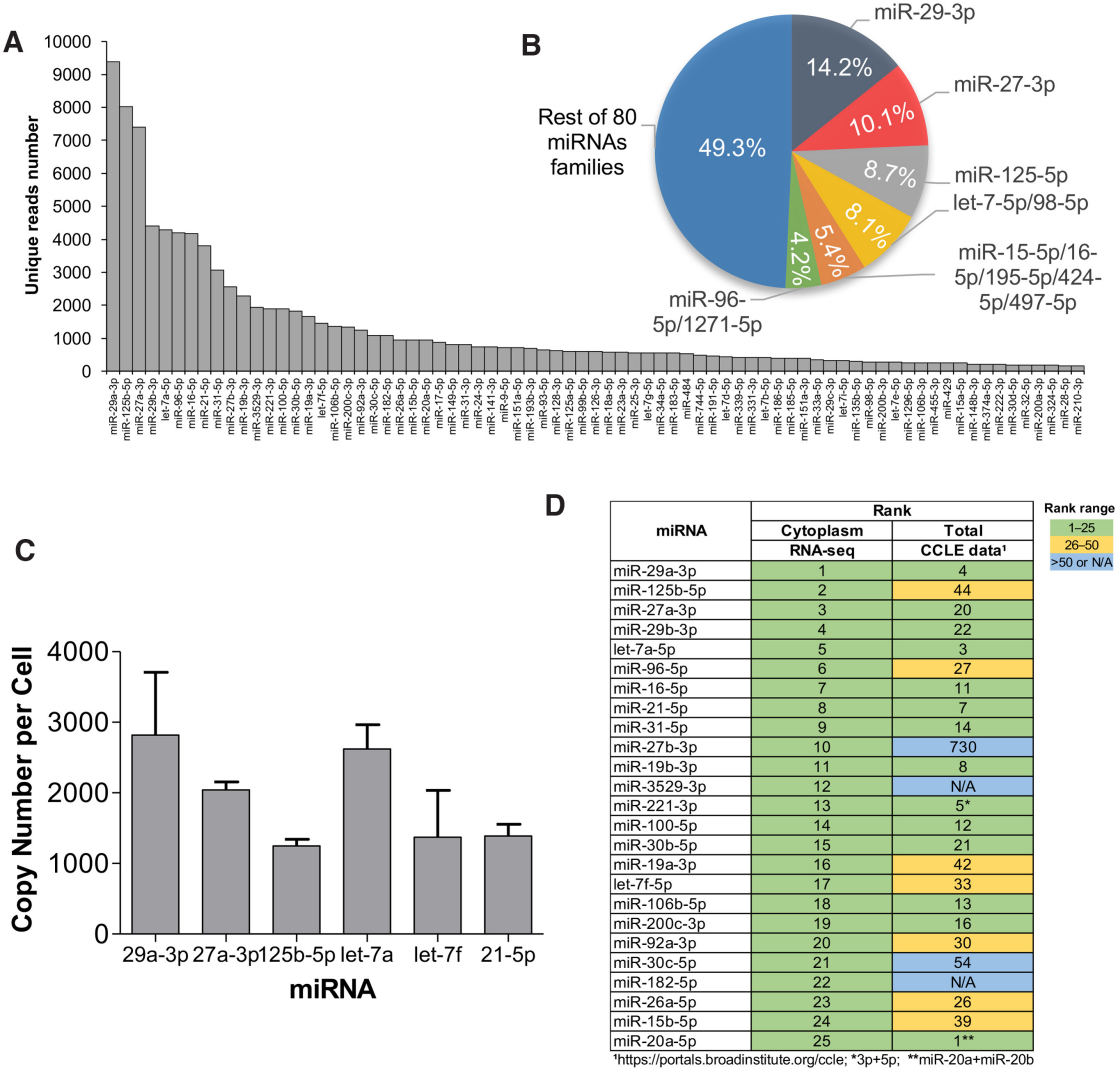
miRNA expression in HCT116 cells. (**A**) miRNA abundance in cytoplasm, ranked by the read number associated with each miRNA after eCLIP. (**B**) Percentages of miRNAs belonging to the six most prevalent families. (**C**) Quantitative PCR (qPCR) determination of copy number of five well-expressed miRNAs. (**D**) Top 25 most prevalent miRNAs in cytoplasm detected by AGO2-eCLIP-seq compared to detection of those miRNAs identified by Nanostring sequencing of HCT116 cells in published CCLE encyclopedia (26). See also Supplemental Figure S3.

We used our eCLIP data to estimate the relative abundance of miRNAs in the cytoplasm of HCT116 cells (Figure 3A). Seventeen miRNAs had over 1000 reads, accounting for >70% of all reads that originate from miRNAs. Half of the top miRNAs reads were associated with just six miRNA families (Figure 3B). Quantitation of miRNA abundance using qPCR revealed that highly expressed miRNAs were present at 1000–3000 copies per cell (Figure 3C). Visual examination of clusters of sequencing reads for top ranked miRNAs confirms unambiguous association of AGO2 and clear discrimination relative to *AGO* knockout cells and input controls (Supplementary Figure S3).

Recently an atlas of steady state miRNA levels was published that used Nanostring sequencing to quantify the relative numbers of miRNAs in hundreds of cell lines (28). Relative to the Illumina RNAseq used in our study, Nanostring uses different probes and detection strategies. While not an exact comparison, the Nanostring data provide a useful benchmark for putting our measured steady state miRNA levels in HCT116 cells into context.

Despite the differences in technologies used for sequencing, our data shared fourteen of the top twenty-five miRNAs with data from the atlas describing miRNAs in HCT116 cells (Figure 3D). Another seven of the top miRNAs from the atlas were in the top fifty miRNAs identified in our data. The similarity between our data and published measurements of miRNA levels supports the belief that HCT116-derived cell lines are good models for probing the action of miRNAs.

### AGO binding and change in steady state mRNA expression

**Figure 4.**
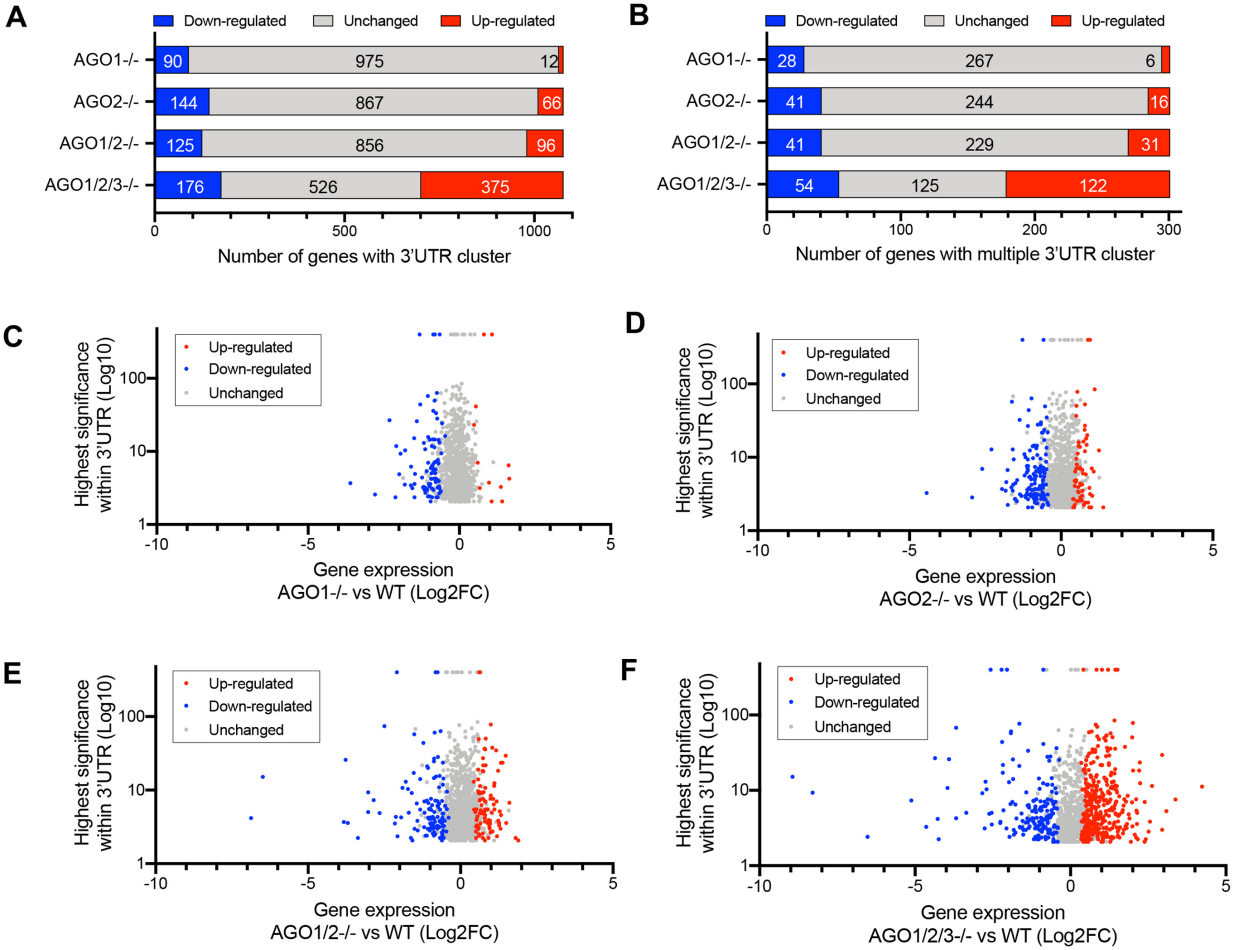
AGO2:3′UTR binding clusters are associated with both up- and down-regulation of gene expression. (**A**) Distribution of gene expression changes for genes with significant AGO2:RNA-binding clusters. (**B**) Distribution of genes with more than one cluster. (**C–F**) Plot of gene expression changes versus cluster significance (**C**) *AGO1*, (**D**) *AGO2*, (**E**) *AGO1/2* and (**F**) *AGO1/2/3* knockout cells. For inclusion in analysis we required peaks to possess a *P* value <0.05 and a >4-fold enrichment in read number for wild-type verse *AGO2* knockouts. See also Supplemental Figure S4.

We performed RNAseq to test how steady state mRNA levels change in *AGO1*, *AGO2*, *AGO1/2* and *AGO1/2/3* knockout cells relative to wild-type cells (Figure 4). Because of the recognized importance of the 3′-UTR, we focused our analysis on genes that possessed significant AGO2 binding sites in that region. Some genes included in our analysis also had AGO2 binding sites in both the coding sequence and the 3′-UTR.

eCLIP revealed that over 1000 genes possessed at least one AGO2-binding sites within their 3′-UTRs (Figure 4A). Three hundred one genes possessed more than one significant AGO2 binding site within their 3′-UTRs (Figure 4B). Contrary to expectations, AGO2 binding sites were associated with both up- and down-regulation of steady state mRNA levels. For *AGO1*, *AGO2* and *AGO1/2* knockout cells more genes with significant clusters showed decreased expression than increased expression relative to wild-type cells (Figure 4A, Supplemental Figure S4A–D). For the *AGO1/2/3* cells, we observed more up-regulated than down-regulated genes. For all knockout cell lines, most genes with AGO2 binding sites showed no significant change in expression.

The general trend is that the number of gene expression changes increase as the expression additional *AGO* genes was knocked out. This result is consistent with the AGO protein variants having redundant functions. This conclusion is also consistent with the data on cell proliferation showing the largest decrease in cell growth for the triple knockout cells (Figure 1E).

We observed little correlation between the magnitudes of change in steady state mRNA levels, the significance of RNAseq reads, and whether gene expression went up, down, or remained relatively unchanged (Figure 4C–F, Supplemental Figure S4E–H). There was no significant correlation for gene activation or gene repression in the *AGO1*, *AGO2* and *AGO1/2* knockout cells. In the *AGO1/2/3* knockout cells there was a slight positive correlation, *R* = 0.19 (*P* < 0.05) for genes with reduced expression and *R* = 0.14 (*P* < 0.05) for genes showing enhanced expression suggesting that AGO2 binding might have a modest impact on gene expression. Absolute changes in gene expression (up or down) were lowest in *AGO1* or *AGO2* knockout cells, greater in *AGO1/2* knockout cells, and greatest in *AGO1/2/3* knockout cells.

### Individual clusters do not predict changes in steady state mRNA levels

**Figure 5.**
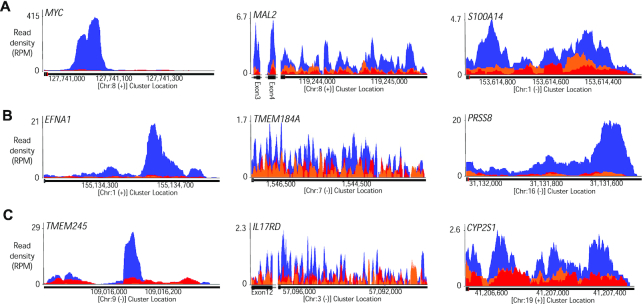
AGO2 binding RNAseq read clusters for genes that are down regulated, do not significantly change, or are up-regulated. Examples of AGO2 binding clusters identified by AGO2 eCLIP-seq. Blue: Wild type cells. Red: *AGO2* knockout cells. Orange: Input control. All clusters are within 3′-UTR regions unless labeled separately (*MAL2, IL17RD*). Representative AGO2-binding clusters for (**A**) down-regulated (*MYC*, *MAL2*, *S100A14*) (**B**) unchanged (*EFNA1*, *TMEM184A*, *PRSS8*) and (**C**) up-regulated genes (*TMEM245*, *IL17RD*, *CYP2S1*). For inclusion in analysis we required peaks to possess a *P* value <0.05 and a >4-fold enrichment in read number for wild-type verse *AGO2* knockouts.

We individually examined clusters of RNAseq reads, ranking them by read number and statistical significance relative to *AGO2* knockout cells (examples shown in Figure 5A–C). After RNAseq, numerical values that characterize clusters are typically presented on a spread sheet and ranked by statistical significance and the fold change in signal above background. It is important, however, to visually inspect data to more fully evaluate the potential for biological significance.

Clusters within 3′-UTRs varied dramatically. Some genes had only one or two clusters. Other genes had multiple clusters in close spatial proximity. The spatial proximity of multiple clusters is likely explained by the observation that more than one AGO protein can bind to the scaffolding protein TNRC6 (37,38). TNRC6 bridges adjacent AGO:miRNA complexes, leading to cooperative interactions that can strengthen binding and activity (39). The presence of adjacent clusters suggests a potential for higher AGO:miRNA occupancy. Greater occupancy at RNA sites would have the potential to have a greater impact on the regulation of gene expression.

The appearance of individual clusters was not a helpful predictor for gene repression, de-repression, or the absence of significant change (Figure 5A–C), with clusters of similar appearance appearing in all three categories. This result is consistent with the lack of a strong correlation between overall cluster significance and steady state mRNA levels (Figure 4). For all three categories (repression/depression/no change), some genes were characterized by a single gene cluster, while others were characterized by multiple clusters.

### Changes in steady state mRNA levels in *AGO1*, *AGO2*, *AGO1/2* and *AGO1/2/3* cells

**Figure 6.**
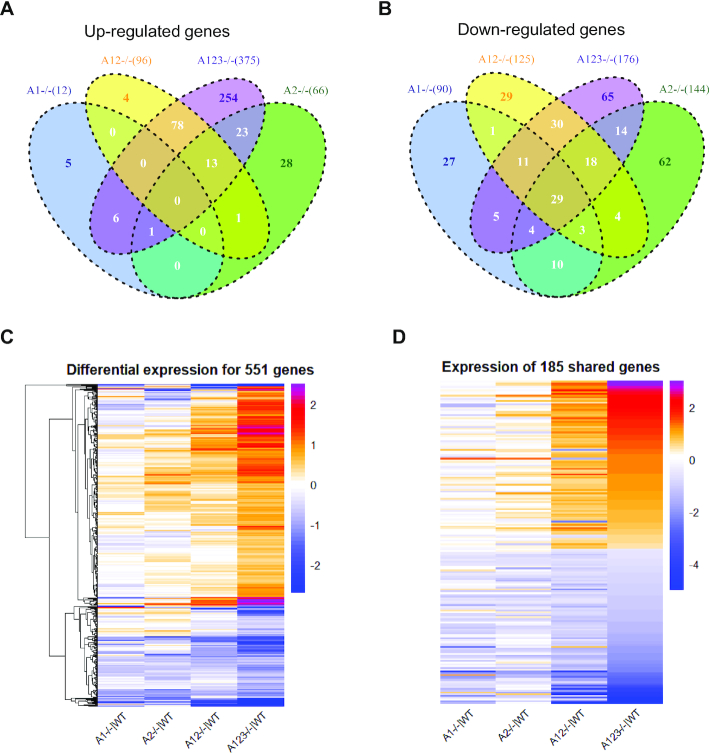
Expression changes in *AGO1*, *AGO2*, *AGO1/2* and *AGO1/2/3* KO cells for genes with AGO2 protein binding clusters within their 3′-UTRs. (**A**, **B**) VENN diagram showing the the overlap among genes with AGO2:3′-UTR-binding sites and significant expression changes relative to wild-type cells for (**A**) increased or (**B**) decreased gene expression in wild-type versus knockout cells. (**C**) Heat map showing how the expression relative to wild-type cells changes in *AGO1*, *AGO2*, *AGO1/2*, and *AGO1/2/3* cells for the 551 genes that have significant AGO protein:3′-UTR clusters and that are differentially expressed in *AGO1/2/3* cells. (**D**) Heat map analysis showing relative changes in gene expression of the 179 AGO-associated genes that show significant expression changes in both *AGO1/2* and *AGO1/2/3* knockout cells.

In *AGO1/2/3* knockout cells, we observe changes in steady state mRNA levels in 551 genes that contain significant RNAseq read clusters (Figure 6A–C). 375 genes were up-regulated (Figure 6A) as would have been predicted by the canonical model, but we also observed that 176 genes were down-regulated (Figure 6B). AGO protein interacts with other RNAi factors including dicer and transactivating response RNA binding protein (TRBP) (40) but we observed little change in the steady state levels of these factors.

For genes that are up-regulated upon knockout of one or more AGO variants, the expression of only seven genes is changed in both the *AGO1* and *AGO1/2/3* knockout cell lines (Figure 6A). For genes that are up-regulated upon knockout of *AGO2*, 37 genes are shared in both *AGO2* and *AGO1/2/3* cells. These results suggest that the *AGO1* and *AGO2* knockouts, individually, have little impact on repression of genes that bind AGO2 within their 3′-UTRs. A much larger number, 91 genes, showed increased expression in both *AGO1/2* and *AGO1/2/3* knockout cells.

For genes that are down-regulated upon knockout of one or more AGO variants, more genes were shared between the *AGO1* or *AGO2* single knockout cells and *AGO1/2/3* triple knockout cells than had been the case for up-regulated genes (Figure 6B). For example, 49 genes were shared between the *AGO1* and *AGO1/2/3* knockout cells, and 65 were shared between *AGO2* and *AGO1/2/3* knockout cells. These data suggest that *AGO1* and *AGO2* expression may have more impact on increasing gene expression than on decreasing gene expression. The expression of 88 genes were changed in both *AGO1/2* and *AGO1/2/3* knockout cells, reinforcing the conclusion that effects become greater as more AGO variants are removed.

We constructed a heat map to visualize the relationship between the changes in steady state mRNA levels for the 551 genes with altered expression (up or down) in the *AGO1/2/3* knockout cells (Figure 6C). The magnitude of change is relatively small in *AGO1* or *AGO2* knockout cells, becomes larger in the *AGO1/2* knockout cells, and reaches a maximum in *AGO1/2/3* knockout cells. Visual inspection suggests that the trends in rank order for expression changes are similar in the four cell lines. For individual genes, most show greater changes in gene expression as more *AGO* variants are knocked out.

The *AGO1/2/3* knockout cells grow more slowly than the other knockout cell lines (Figure 1E). Slower growth raises the possibility that there are more widespread changes in gene expression that could indirectly affect expression of genes that contain AGO2-binding sites. By contrast, the growth of *AGO1/2* knockout cells was more similar to wild-type. Therefore, we focused on the 179 genes that are altered in both *AGO1/2* and *AGO1/2/3* knockout cells. These 179 genes have a higher potential to be more directly related to a knockout of AGO protein rather than a more indirect effect from global gene changes that manifest in slower cell growth.

Data on steady state mRNA levels in *AGO1/2* and *AGO1/2/3* knockout cells revealed a strong correlation between the gene expression changes observed for individual genes in the two cell lines (Figure 6D). While the magnitude of changes for *AGO1* and *AGO2* knockout cells are less, there is a strong trend among all knockout *AGO* knockout cell lines separating down-regulated and up-regulated genes. These data reinforce the conclusion that AGO proteins exert similar effects and that those effects become more pronounced as the combined level of AGO protein is reduced.

### Impact of AGO2:3′-UTR association on mRNA levels for individual genes

**Figure 7.**
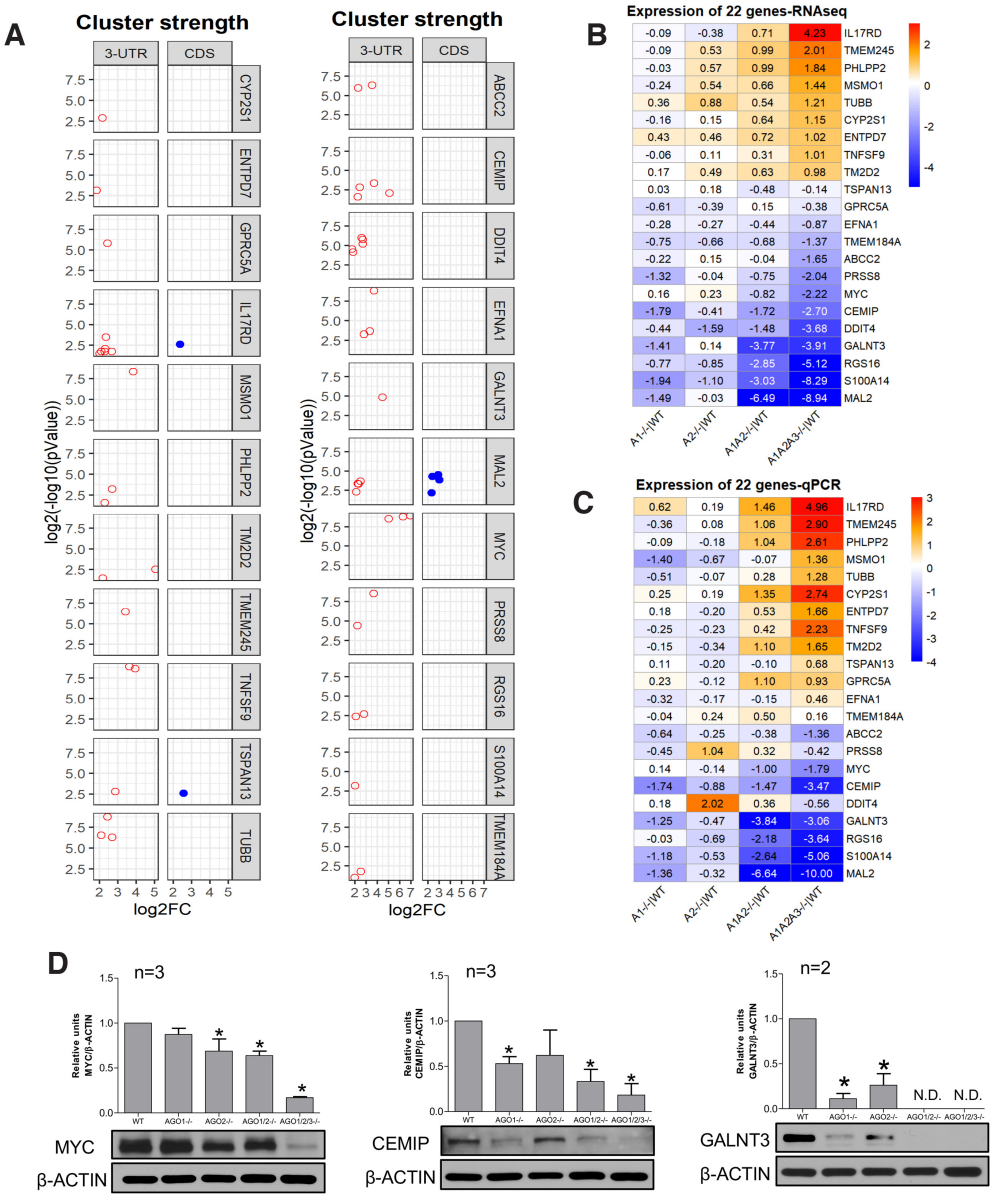
Impact of engagement with AGO on the expression of 22 representative genes. (**A**) Cluster strength, significance, and location. (**B**) Expression level relative to wild type for 22 selected genes, evaluated by RNAseq and ranked highest to lowest based on the changes in *AGO1/2/3* knock out cells relative to WT. (**C**) Expression level relative to wild type for 22 selected genes, evaluated by qPCR. Ordering is based on RNAseq rankings. Values for B and C are log_2_(fold change) for knockout versus wild-type cells. (**D**) Western analysis of MYC, CEMIP and GALNT3 in wild type and knockout cells. *P* < 0.05: * compare to WT. See also Supplemental Figure S5.

We chose 22 genes for detailed analysis of mRNA levels. Genes were chosen based on the presence of at least one highly significant cluster of RNAseq reads within the 3′-UTR (Figure 7A, Supplemental Figure S5A-C). Three genes (*IL17RD*, *TSPAN13*, *MAL2*) also had one or more AGO2 binding sites within the coding region near the 3′-UTR. Eighteen of the twenty-two genes had at least one calculated site of seed-sequence complementarity for a miRNA ranked in the top 25 for prevalence by RNAseq (Figure 3, Supplemental Figure S5D). Several had multiple sites for seed sequence complementarity with highly ranked miRNAs.

As we had observed for the entire group of genes that contain binding sites for AGO protein (Figure 4), for the 22 selected genes the knockout of *AGO* expression led to a variety of outcomes—gene repression, de-repression, or the absence of significance change (Figure 7B, Supplemental Figure S5A–C). The changes in the *AGO1* or *AGO2* knockout cells are relatively small. While we did not obtain *AGO3* knockout cells, we did knock AGO3 expression down using siRNAs complementary to *AGO3* mRNA (Supplemental Figure S6). As with the AGO1 or AGO2 single knockouts, the changes in RNA levels upon knocking down only AGO3 were relatively small. Changes in steady state mRNA levels are larger in the *AGO1/2* knockout cells and maximal in the *AGO1/2/3* cells.

Genes that were more repressed or activated in *AGO1/2* knockout cells tended to also be among the most repressed or activated genes in *AGO1/2/3* knockout cells. The trend for the magnitude of gene expression to be greater in *AGO1/2* and *AGO1/2/3* knockout cells versus *AGO1* or *AGO2* single knockouts is consistent with the larger number of genes that show significantly altered expression (Figures 4 and 5). In Figure 7B, changes are reported in log_2_ fold scale. In absolute values, the change in expression for *AGO1/2/3* knockout cells ranged from and 18-fold increase for *IL17D* expression to almost complete repression for expression of *MAL2*.

We crosschecked the RNAseq data for the 22 genes using quantitative PCR (qPCR) (Figure 7C, Supplemental Figure S5E). As observed for RNAseq, The expression changes were smallest for the *AGO1* and *AGO2* single knockout cells. The qPCR data for *AGO1/2* and *AGO1/2/3* knockout again showed the greatest changes. The rank order of expression changes observed by qPCR was similar to that observed in our RNAseq data (Figure 7B).

We analyzed protein expression to determine whether changes in RNA levels were predictive of changes in protein expression (Figure 7C, Supplemental Figure S5F). Genes were chosen based on the availability of antibodies adequate for western analysis. We observe decreases in MYC, CEMIP and GALNT3 protein expression as had been predicted by RNAseq and qPCR. Effects on protein expression were greatest in the *AGO1/2/3* knockout cells, consistent with RNA measurements.

## DISCUSSION

### Reevaluating the standard model linking AGO binding to gene repression

The premise underlying the standard model for the action of miRNAs is that miRNA:AGO complexes recognize sequences within the 3′-UTR. This recognition is primarily through ‘seed sequence’ pairing of bases 2–7 at the 5′ end of miRNAs, with the potential for other bases to contribute (8,41). Recognition is associated with translational repression, with hundreds or thousands of genes predicted to be subject to this repressive mechanism (42). Repression is often inferred based on seed sequence complementarity to an mRNA of interest, helping shape the hypotheses of thousands of publications each year.

In accord with these common assumptions, the detection of AGO binding by CLIP within a 3′-UTR should be a major factor predicting gene repression by miRNAs. Our goal was to test this hypothesis by combining *AGO* knockout cells, RNAseq, and anti-AGO2 eCLIP (Figure 1), followed by analysis of the linkage between AGO2 binding within 3′-UTRs and altered gene expression.

Our experiments were performed in only one cell line, HCT116. HCT116, however, appears to be a typical cell line with regards to miRNA expression (28) and has been the subject of hundreds of studies involving miRNAs and potential biological interactions. It is possible that correlations between AGO2 binding sites and steady state RNA levels of target genes may be different in other cell lines, cells grown under differ conditions, or cell lines growing *in vivo*. It may be necessary to establish correlations on a case-by-case basis rather than assuming an association between binding and gene repression.

### Redundant roles for *AGO1*, *AGO2* and *AGO3* governing gene expression

We evaluated gene steady state mRNA levels in *AGO1*, *AGO2*, *AGO1/2* and *AGO1/2/3* knockout cells. Trends for expression of genes relative to one another are similar in all knockout cell lines (Figures 6C and D, 7B and C), consistent with AGO protein variants performing similar functions.

While trends are similar, the absolute levels of gene expression change at the individual genes vary dramatically. The effects of *AGO* knockouts are relatively small in the *AGO1* or *AGO2* single knockouts, become more pronounced in the double knockout *AGO1/2* cells, and become most extreme in the *AGO1/2/3* knockout cells. These data are consistent with the observation that cell growth of the *AGO1/2/3* knockout cells was slowest (Figure 1E).

While our data do not exclude the possibility that the AGO1, AGO2 or AGO3 protein variants have unique functions, the data suggest that AGO1, AGO2 and AGO3 have redundant functions that tend to push the expression of genes in similar directions. Overlapping functions for AGO variants has previously been suggested (12–14,43,44). FLAG-HA-tagged AGO1-4 bind to similar sites within the transcriptome (31) supporting the conclusion that endogenously expressed AGO1 and AGO3 proteins will also bind to sites like the ones where we observe AGO2 association.

### AGO:3′-UTR binding is associated with gene repression and de-repression

It would have been unrealistic to expect that knockout of AGO proteins would only result in de-repression of genes (45). A change in the expression of one gene will inevitably produce changes in the expression of other genes that are not directly due to association of AGO protein within the gene's 3′-UTR. These indirect secondary effects on gene expression would be expected to have a confounding impact that would confuse analysis. Nevertheless, based on standard assumptions that miRNA:AGO complexes bind 3′-UTRs and repress gene expression, we had assumed that the strength and significance of AGO protein binding would be an important factor predicting gene de-repression upon knocking out *AGO* gene expression.

Our data do not reveal a predictive link between AGO2 protein binding and gene repression (Figures 4–7). Some genes with significant AGO2 binding sites within their 3′-UTRs showed the increased expression – the expected outcome. However, for *AGO1*, *AGO2* and *AGO1/2* knockout cells, more genes showed an unexpected result - decreased expression.

For *AGO1/2/3* knockout cells, more genes showed increased expression, as might have been predicted by the standard model. Even for these triple knockout cells, however, we observed that expression of many genes decreased. Also, most genes with AGO:3′-UTR-binding sites did not exhibit changes in expression that met a minimum threshold for up- or down- regulation. There is little predictive correlation between the significance or magnitude of AGO2-binding within 3′-UTRs and gene repression or de-repression. Consistent with the lack of obvious predictive insight from global analysis of gene expression changes, inspection of individual genes with AGO2 binding sites also failed to show any features distinguishing the expected outcome of gene repression from gene activation or lack of significant change (Figure 5, Supplemental Figure S5).

Previous work has suggested that mRNA levels are correlated with protein levels but that this correlation can be affected by growth conditions or other factors (46). Uncertainty about the correlation between steady state RNA and protein levels requires another layer of caution when interpreting the significance of RNAseq data.

### *MYC*: Strong AGO:3′-UTR interaction is associated with gene activation

*MYC* is an oncogene whose overexpression can lead to cancer (47). Several previous studies have concluded that *MYC* expression can be down-regulated by miRNAs (48–50). Our eCLIP data showed that the strongest read cluster other than direct binding to miRNAs was located within the *MYC* 3′-UTR. *Let-7* is one of the most highly expressed miRNAs in HCT116 cells, and there is a strong let-7 site within the *MYC* 3′-UTR (Supplemental Figures S5D and S7).

The simplest conclusion from combining our eCLIP data with standard assumptions about miRNAs, the existence of a likely *Let-7* target site, and the previous reports of miRNA action would be that *MYC* expression is repressed by miRNAs in HCT116 cells. This conclusion would then lead to the hypothesis that the repression might be important for understanding the regulation of *MYC* expression during cancer.

However, when we correlated the association of AGO2 with the difference in expression of *MYC* for wild-type versus *AGO1/2/3* knockout cells, we observed the opposite result. In *AGO1/2/3* knockout cells, the expression of *MYC* RNA and protein decreases relative to wild-type cells. Rather than repress *MYC* expression, our data suggest that the RNAi machinery promotes expression.

Whether this regulation of *MYC* gene expression is direct or indirect is not clear and conclusively determining the mechanism is beyond the scope of this study. Increased expression might be due to a non-canonical mechanism in which a miRNA binds the 3′-UTR and activates gene expression (51). *MYC* is a highly expressed gene, so it is also possible that it might act as a miRNA ‘sponge’ to attract enough miRNAs to affect global regulatory pathways. Alternatively, knocking out *AGO* may trigger changes in gene expression that indirectly drive *MYC* levels higher.

Regardless of the mechanism responsible for reduced *MYC* expression, the simple assumption from our eCLIP data, that *MYC* expression is repressed by a miRNA:AGO complex, is incorrect. *MYC* is a critical gene for understanding cancer. Making progress towards understanding the potential for miRNAs to control *MYC* and other genes underlying disease will benefit from reexamining prior assumptions.

## CONCLUSIONS

The control of mammalian gene expression by miRNAs has been the subject of intense interest for almost twenty years. While the interplay of RNA and gene expression is known to be a complex phenomenon (52), many reports have revolved around the simple assumption that recognition by AGO:miRNA complexes leads to repression of gene expression.

Contrary to these assumptions, AGO-bound sequences within 3′-UTRs do not appear to be reliable predictors of gene repression. If eCLIP data do not correlate well with the potential for miRNA-mediated gene repression, studies that rely only on computationally predicted seed matches for miRNAs will be even more problematic. Our data do not dispute the canonical mechanism that AGO:miRNA complexes can bind to 3′-UTRs and repress gene expression. That conclusion has been amply proven by many studies using 3′-UTRs engineered to include binding sites for miRNAs (2). Our data do suggest that inferring an association between repression of endogenous gene expression and binding of miRNA:AGO complexes at a site is not straightforward. Gene repression based on seed sequence matches should not be assumed in the absence of mechanistic biochemistry.

Our findings raise questions about the origins and mechanistic significance of the sites for AGO:3′-UTR binding that are not associated with gene repression. What is the role of AGO binding? Can AGO be involved in gene activation? Answering these questions, and the further questions that will arise from them, will be required to better understand the basic science of miRNA action and broaden options for therapeutic development. The standard mechanism should not be viewed as the only possible mechanism for the action of miRNAs. AGO may regulate the action of target RNAs through non-canonical mechanisms beyond repression of translation in cell cytoplasm.

## DATA AVAILABILITY

All high-throughput sequencing data generated in the course of this study (RNAseq, eCLIP) have been deposited in Gene Expression Omnibus under accession number GSE146688.

## Supplementary Material

gkaa478_Supplemental_File
