# Supplementary material for: Argonaute binding within 3′-untranslated regions poorly predicts gene repression
Source: Nucleic Acids Res. 2020 Jun 5;48(13):7439–53. doi: 10.1093/nar/gkaa478 (PMC7367155; doi:10.1093/nar/gkaa478)
Supplement: gkaa478_Supplemental_File [file gkaa478_supplemental_file.pdf]

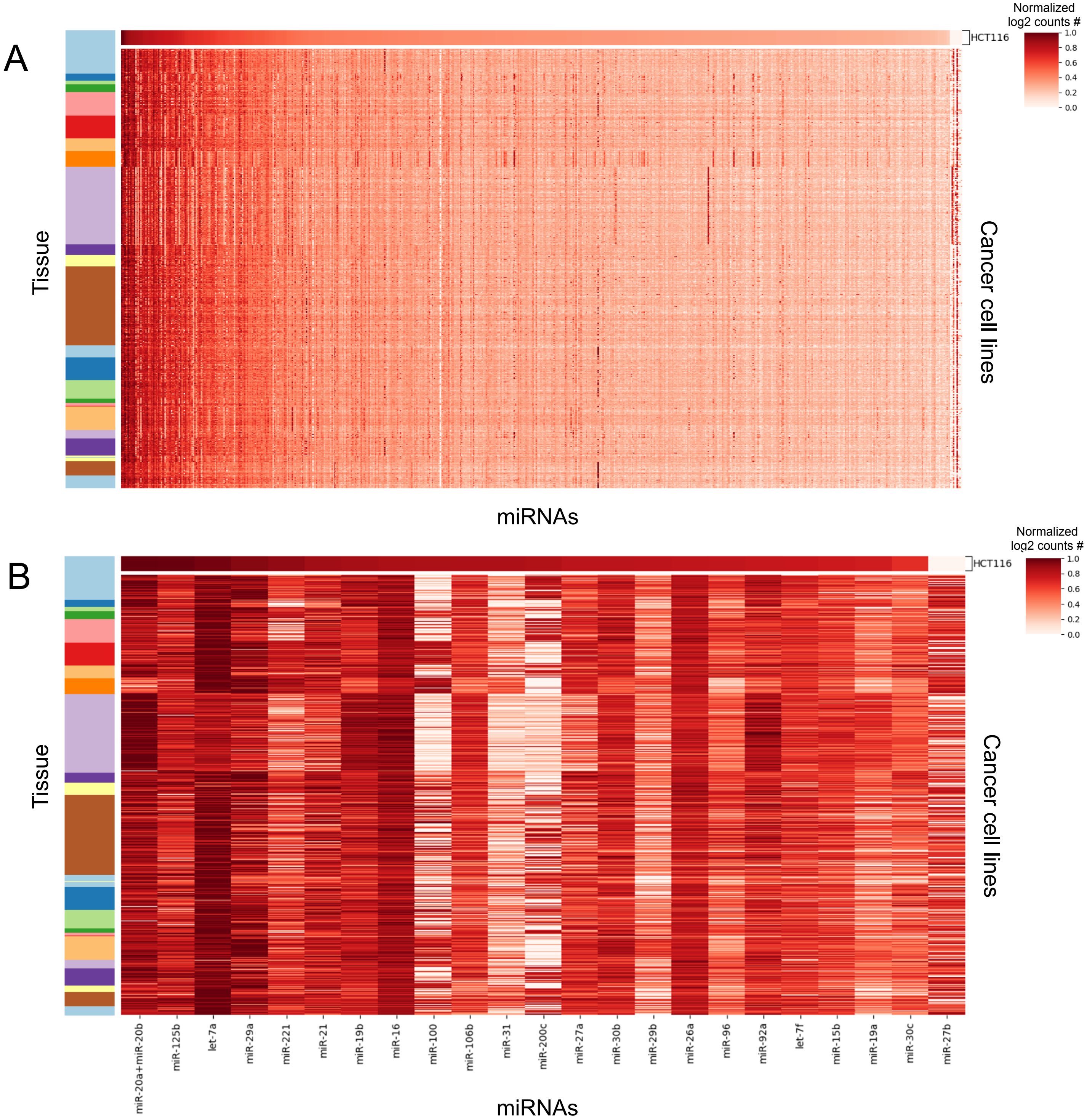

Figure S1 (related to Figure 1). miRNA expression profile in cancel cells based on Cancer Cell Line Encyclopedia (CCLE). The expression of each miRNA in HCT116 cells (from highest to lowest) is depicted on the top line. miRNAs that are more highly expressed in HCT116 cells tend to be more highly expressed across a broad panel of cell lines. For consistency, all data are published nanonstring data (Ghandi et al., 2019). (**A**) Relative expression of miRNAs in cancer cell lines. (**B**) Relative expression of the most highly expressed miRNAs in HCT116 cells and other cell lines. Tissue color bar order from top to bottom (LARGE INTESTINE, AUTONOMIC GANGLIA, BILIARY TRACT, BONE, BREAST, CNS, ENDOMETRIUM, FIBROBLAST, HAEMATOPOIETIC AND LYMPHOID, KIDNEY, LIVER, LUNG, OESOPHAGUS, OVARY, PANCREAS, PLEURA, PROSTATE, SALIVARY GLAND, SKIN, SMALL, SOFT TISSUE, STOMACH, THYROID, UPPER AERODIGESTIVE TRACT, URINARY TRACT).

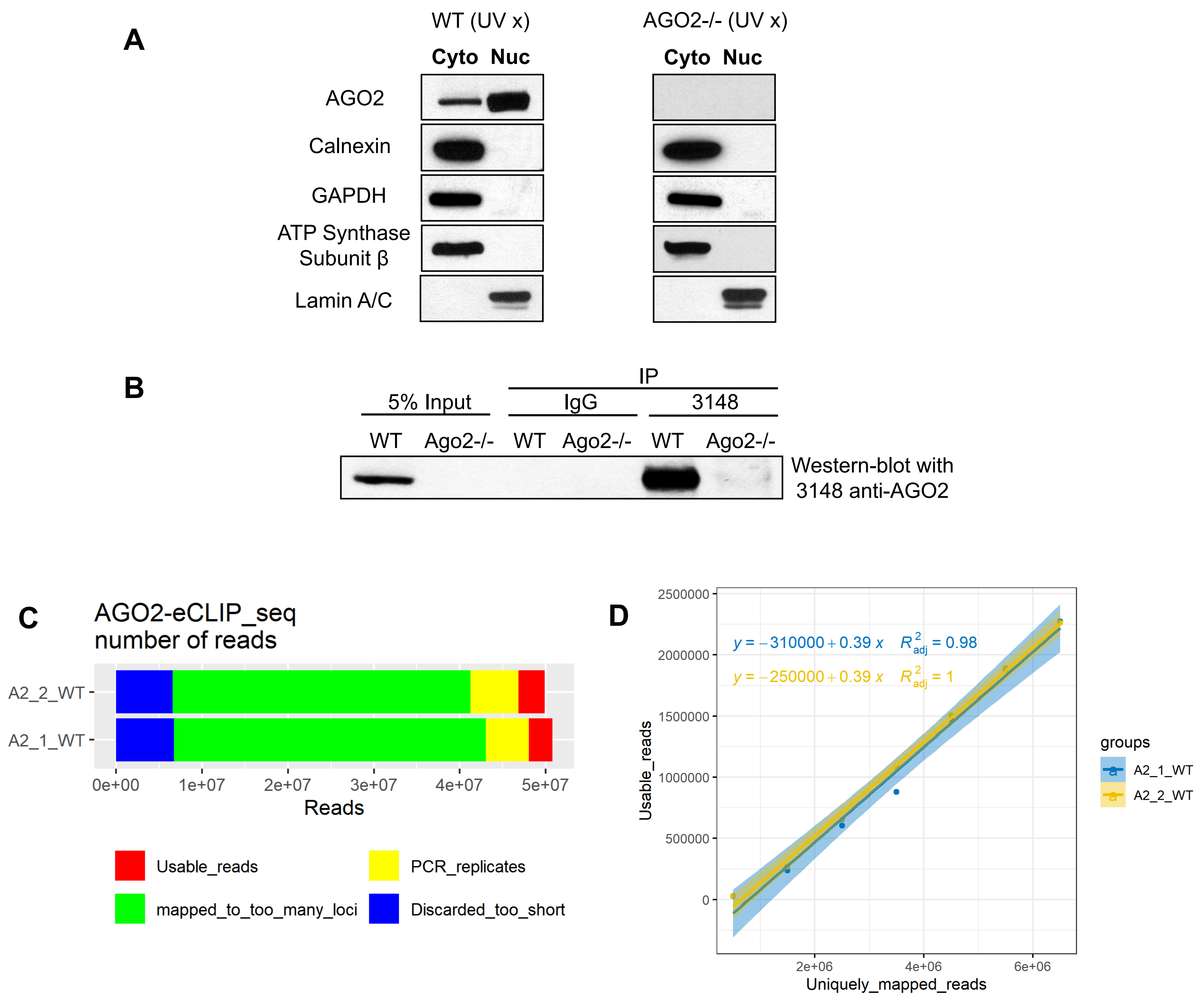

Figure S2 (Related to Figure 2). Purity of sample preparation and immunoprecipitation. (**A**) Western blot of wild-type and *AGO2* knock out cells showing the purity of cytoplasm prep for eCLIP. Calnexin is a marker for the endoplasmic reticulum, a structure contiguous with the nuclear membrane that must be removed to ensure adequate nuclear purification. GAPDH and ATP synthase beta are markers for cytoplasm. Lamin A/C is a nuclear marker that should be absent if a cytoplasmic sample is pure. and (**B**) Pull down using anti-AGO2 rabbit polyclonal antibody (3148) relative to input sample (no Ig pulldown) and a sample treated with a non-cognate control antibody.

Analysis of anti-AGO2 eCLIP-seq biological replicates showing comparable data quality. (**C**) Sequencing reads distribution, showing the total number of usable reads. (**D**). Correlation between uniquely mapped reads and usable reads. Different number of uniquely aligned reads were randomly sampled from AGO2 eCLIP experiments and PCR replicates were removed. Points indicate the mean of 10 sampling experiments.

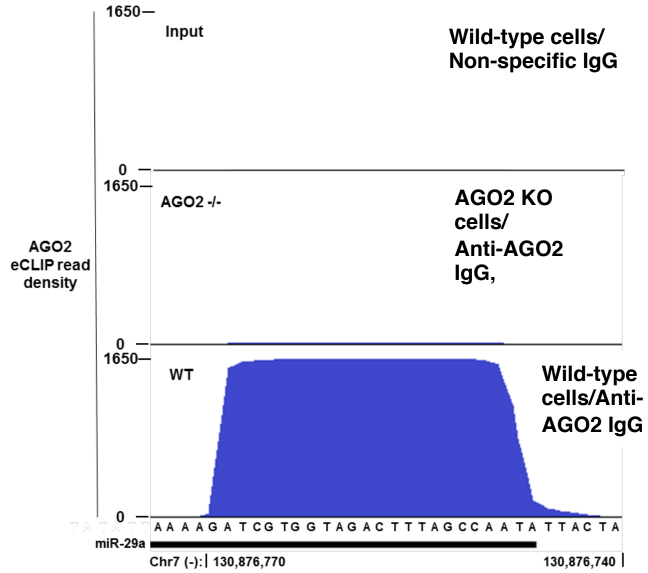

Figure S3 (Related to Figure 3). Sample eCLIP cluster data for highly ranked cluster – miR-29a. Three cytoplasmic extract samples were analyzed by RNAseq: 1) extract from wild-type cells that had been treated with a non-specific antibody (IgG); 2) extract from *AGO2* knockout cells that had been treated with anti-AGO2 antibody; and 3) extract from wild-type cells that had been treated with anti-AGO2 antibody. This is typical eCLIP data characterizing miRNA engagement and shows the high signal to noise routinely observed when detecting AGO2:miRNA association.

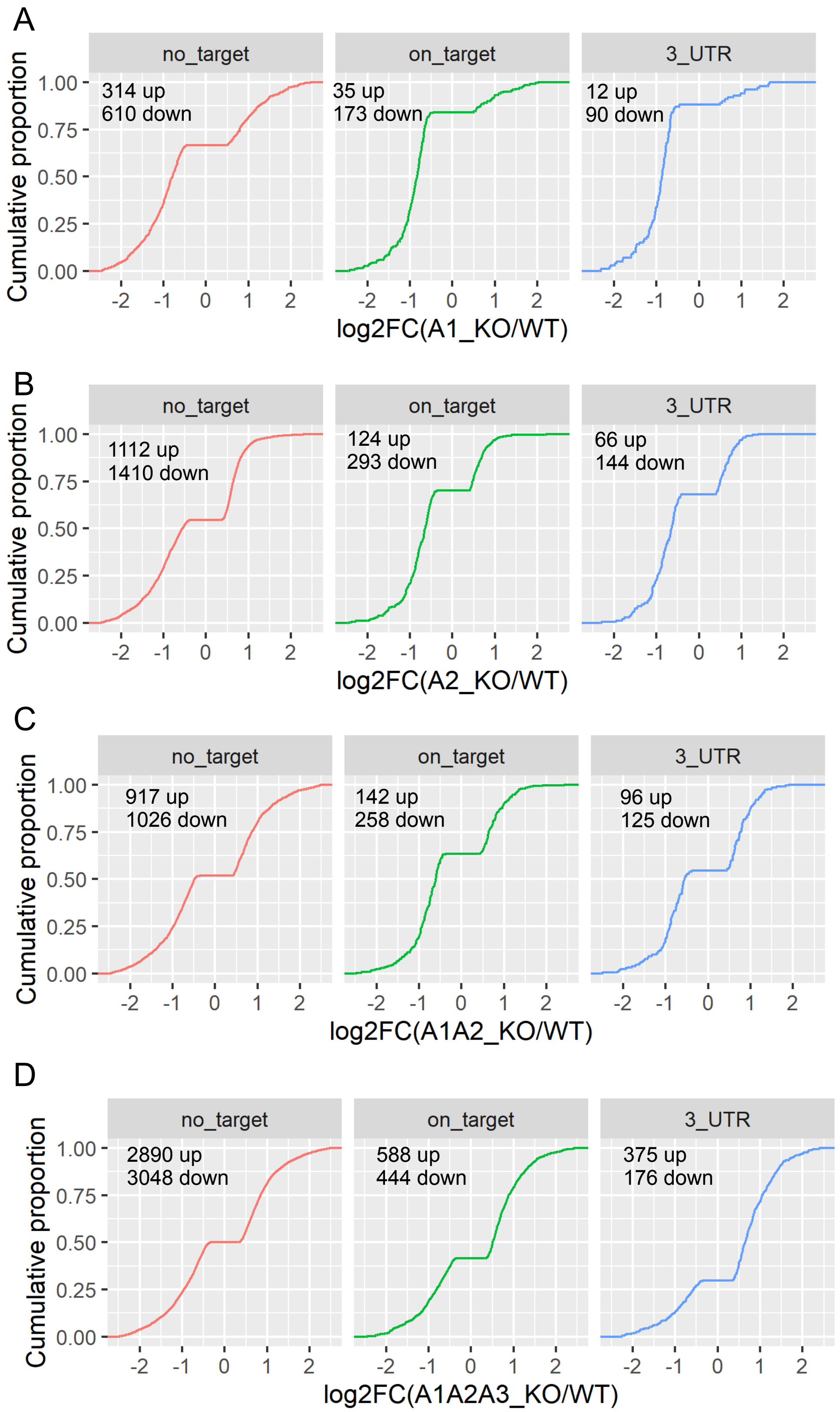

Supplemental Figure S4 (Related to Figure 4). The effects of *AGO1*, *AGO2*, *AGO1/2*, and *AGO1/2/3* knockouts on gene regulation in HT116 cells. CDF plots of log2 fold change upon *AGO* knockout for genes having no AGO2-binding clusters detected at any location, genes that have AGO2 clusters within their mRNAs, and genes that have AGO2 clusters with their 3'-UTRs. (**A-D**) Data for *AGO1*, *AGO2*, *AGO1/2*, and *AGO1/2/3* knockout cells respectively. The total number of genes that are significantly up- or down- regulated are noted.

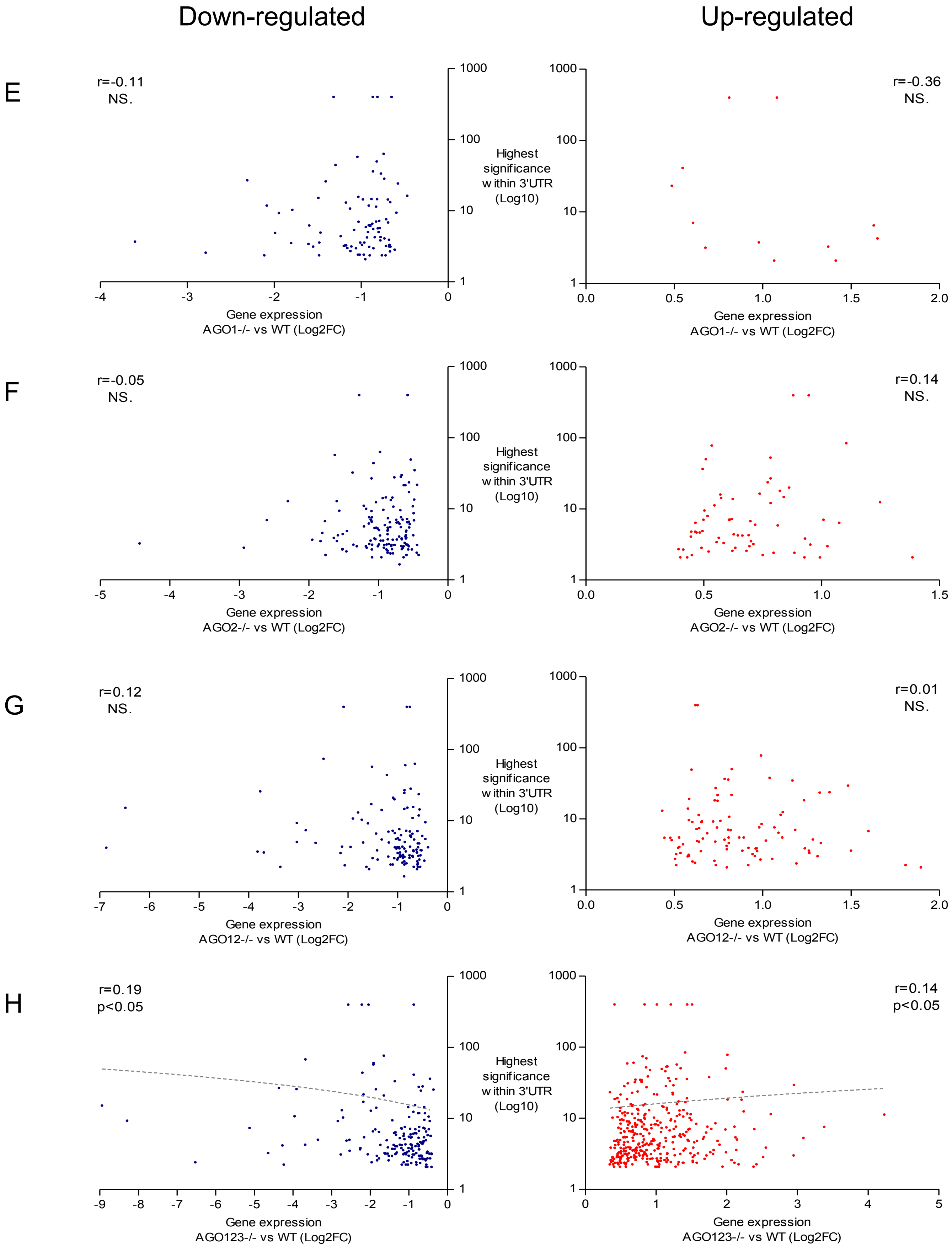

Supplemental Figure S4 (Related to Figure 4). Correlation between significance of AGO2:3'-UTR binding clusters and gene expression change in *AGO* KO cell lines. (**E-H**) Data for *AGO1*, *AGO2*, *AGO1/2*, and *AGO1/2/3* knockout cells respectively. Blue: downregulated genes. Red: Up-regulated genes. The data do not pass the normality test for both data sets, or relation was not linear, therefore Spearman correlation was applied. $p<0.05$ was only achieved in *AGO1/2/3* knockout datasets.

Supplemental Figure S5. Appearance of clusters for 22 representative genes. (A) Clusters that are associated with increased gene expression as determined by RNAseq. (**B**) Clusters that are not associated with significant changed. (**C**) Clusters that are associated with reduced gene expression.

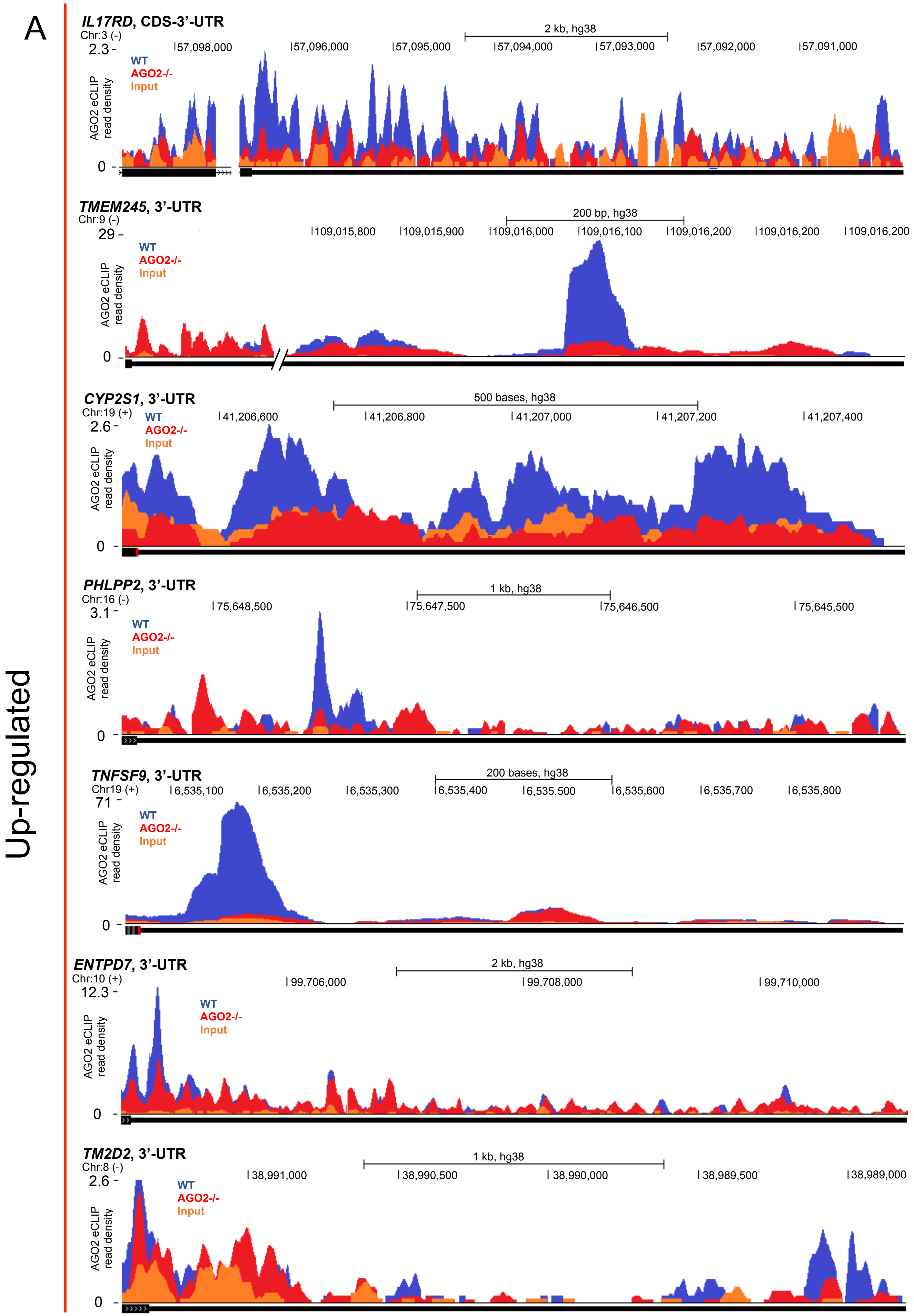

Supplemental Figure S5. Appearance of clusters for 22 representative genes. (A) Clusters that are associated with increased gene expression as determined by RNAseq. (**B**) Clusters that are not associated with significant changed. (**C**) Clusters that are associated with reduced gene expression.

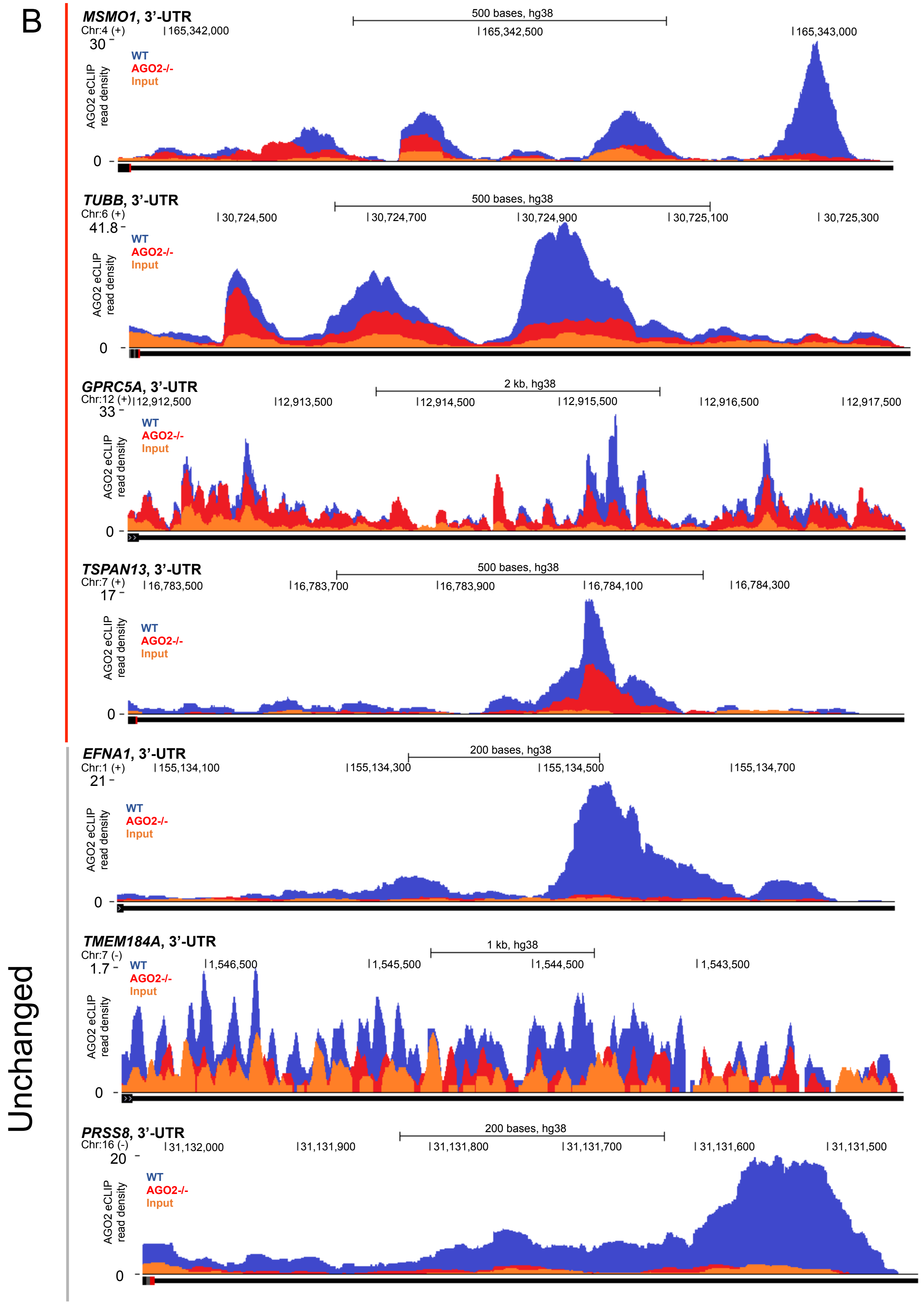

Supplemental Figure S5. Appearance of clusters for 22 representative genes. (A) Clusters that are associated with increased gene expression as determined by RNAseq. (**B**) Clusters that are not associated with significant changed. (**C**) Clusters that are associated with reduced gene expression.

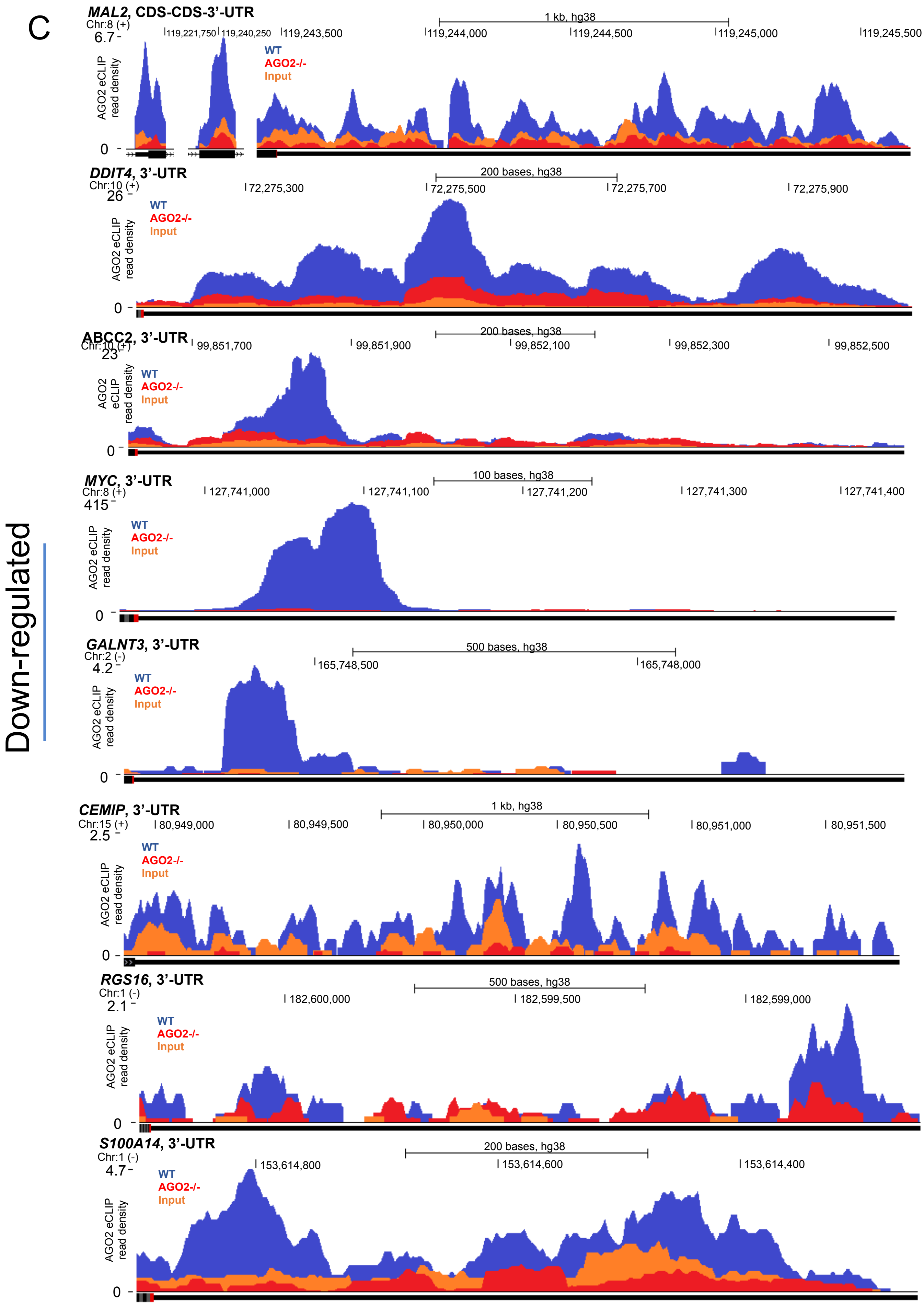

Supplemental Figure S5D. Predicted candidates sites and complementarity for miRNA engagement with 22 representative genes. Candidate binding sites outlined in red are potential sites for binding to the experimentally determined (Figure 3) most prevalent 25 miRNAs in HCT116 cells.

IL17RD

Chr3: 57,096,218-57,096,355

```
     3' gaCGUCUUGACAAGGGCGACGAu 5' hsa-miR-503
          |||  :|   |:: ||||||
  17:5' aaGCAUUGCCACUUUAGCUGCUg 3' IL17RD

     3' ucgcccugaaACUCCCGGUCAa 5' hsa-miR-193b
                  ||  |||||||
 114:5' uggagugaaaUGCUGGCCAGUa 3' IL17RD

     3' ucgauacggucguaGAACGGa 5' hsa-miR-31
                      ||||||
 132:5' aguacuuguucuccCUUGCCc 3' IL17RD

     3' aguccuugacggaaaGAGAGGu 5' hsa-miR-185
                       ||||||
 165:5' ggauaucuugacaaaCUCUCCa 3' IL17RD
```

Chr3: 57,096,147 57,096,190

```
     3' ucgAUACGGUCGUAGAACGGa 5' hsa-miR-31
           | || || || ||||||
 228:5' aggUCUGACAACAGCUUGCCa 3' IL17RD

     3' ucacACUCAAGA-UG--GU--AACGGUUu 5' hsa-miR-182
            | || ||| ||  ||  |||||||
 222:5' uccaUAAGGUCUGACAACAGCUUGCCAAa 3' IL17RD

     3' ucguuuuuacacgaucACGGUUu 5' hsa-miR-96
                        ||||||
 228:5' aggucugacaacagcuUGCCAAa 3' IL17RD
```

Chr3: 57,095,954-57,095,993

```
     3' gauggacgugacaUUCGUGAAAc 5' hsa-miR-17
                     |:|||||||
 398:5' gcuuugugaaaaaAGGCACUUUu 3' IL17RD

     3' gauGGACG-UGAUAUUCGUGAAAu 5' hsa-miR-20a
           ::||: |  | |:|||||||
 397:5' ugcUUUGUGAAAAAAGGCACUUUu 3' IL17RD

     3' uagacgugacagUCGUGAAAu 5' hsa-miR-106b
                    :|||||||
 400:5' uuugugaaaaaaGGCACUUUu 3' IL17RD

     3' gauggacgugacaUUCGUGAAAc 5' hsa-miR-17
                     |:|||||||
 398:5' gcuuugugaaaaaAGGCACUUUu 3' IL17RD

     3' ucgccCUGAAACUCCCGGUCAa 5' hsa-miR-193b
             ||  |  || ||||||
 431:5' ccacaGAAAUCAAGUGCCAGUc 3' IL17RD
```

Chr3:57,095,180-57,095,236

```
     3' ggguUGUUGUACUUUGAUGGAu 5' hsa-miR-196a
            ||| | |   |||||||
1200:5' guguACAUC-UCUCACUACCUu 3' IL17RD

     3' ggguuguuguccuuUGAUGGAu 5' hsa-miR-196b
                      |||||||
1199:5' uguguacaucucucACUACCUu 3' IL17RD
```

Chr3: 57,095,034-57,095,070

```
     3' cgCGGGU--UAAUU--ACAGACAACUa 5' hsa-miR-421
          | |||  || ||   || ||||||
1302:5' aaGACCAACAUGAAGGGGUAUGUUGAg 3' IL17RD
```

Chr3: 57,094,870-57,094,921

```
     3' uguCAAGAAGUUGACCGUCGAa 5' hsa-miR-22
           | ||| :||  |||||||
1471:5' acuGCUCUGUAA-AGGCAGCUg 3' IL17RD

     3' ugucaagaaGUUGACCGUCGAa 5' hsa-miR-22
                 ||:|||||||||
1477:5' cuguaaaggCAGCUGGCAGCUu 3' IL17RD

     3' agcgggagaGUUGG--GUCGAAAa 5' hsa-miR-320a
                 ||:|:  |||||||
1477:5' cuguaaaggCAGCUGGCAGCUUUu 3' IL17RD
```

TMEM245/C9orf5

Chr9: 109,015,437-109,015,517

```
     3' ugggucaucggucuACAUCGa 5' hsa-miR-222
                      ||||||
4964:5' gauguaacucucauUGUAGCc 3' C9orf5

     3' aguguauccuuacuuuUCGGUAu 5' hsa-miR-135b
                        ||||||
4965:5' auguaacucucauuguAGCCAUc 3' C9orf5

     3' aguaugucgaucuauuGGUUUCu 5' hsa-miR-9
                        ||||||
4972:5' ucucauuguagccaucCCAAAGa 3' C9orf5

     3' ucGGGUUU-UCCUCUUAAGAAAc 5' hsa-miR-186
          |||||| |  |   ||||||
4985:5' auCCCAAAGAAAACCCUUCUUUg 3' C9orf5
```

PHLPP2

Chr16:71,647,901-71,647,987

```
     3' cgaaacuguuaugauaACGUGAc 5' hsa-miR-301a
                        ||||||
 897:5' uuuuuauuuuugaaucUGCACUc 3' PHLPP2

     3' ugggauauucguuauaACGUGAu 5' hsa-miR-454
                        ||||||
 897:5' uuuuuauuuuugaaucUGCACUc 3' PHLPP2

     3' gacAAGGAC---GACUUGA-CUCGGu 5' hsa-miR-24
           ||::||   ||| ||| |||||
 900:5' uuaUUUUUGAAUCUGCACUCGAGCCa 3' PHLPP2

     3' gauagacgugaucuaCGUGGAAu 5' hsa-miR-18a
                       |||||||
 931:5' cuuagaggcagcccgGCACCUUc 3' PHLPP2
```

TNFSF9

Chr19: 6,535,114-6,535,241

```
     3' auuggcuaaagucuACCACGAu 5' hsa-miR-29a
                      |||||||
  53:5' uucauggagaccccUGGUGCUg 3' TNFSF9

     3' uugugacuaaaguuuACCACGAu 5' hsa-miR-29b
                       |||||||
  52:5' cuucauggagaccccUGGUGCUg 3' TNFSF9

     3' auuggcuaaaguuuACCACGAu 5' hsa-miR-29c
                      |||||||
  53:5' uucauggagaccccUGGUGCUg 3' TNFSF9

     3' gacGUCUUGA--CAAGGGCGACGAu 5' hsa-miR-503
           ::| :||  || ||:||||||
  64:5' cccUGGUGCUGGGUCCCUGCUGCUu 3' TNFSF9

     3' guguuugguaauacACGACGAu 5' hsa-miR-15a
                      |||||||
  67:5' uggugcugggucccUGCUGCUu 3' TNFSF9

     3' aaguuuuguacuuaACGACGAc 5' hsa-miR-424
                      |||||||
  67:5' uggugcugggucccUGCUGCUu 3' TNFSF9

     3' acauuugguacuacACGACGAu 5' hsa-miR-15b
                      |||||||
  67:5' uggugcugggucccUGCUGCUu 3' TNFSF9

     3' gcgguuauaaAUGCACGACGAu 5' hsa-miR-16
                  | | |||||||
  67:5' uggugcugggUCCCUGCUGCUu 3' TNFSF9

     3' guguuugguaauacACGACGAu 5' hsa-miR-15a
                      |||||||
 106:5' uuggcaggggucccUGCUGCUg 3' TNFSF9

     3' aaguuUUGUACUUA---ACGACGAc 5' hsa-miR-424
             ::|| |::|   |||||||
 103:5' ggcuuGGCAGGGGUCCCUGCUGCUg 3' TNFSF9

     3' acauuugguacuacACGACGAu 5' hsa-miR-15b
                      |||||||
 106:5' uuggcaggggucccUGCUGCUg 3' TNFSF9

     3' gcgguuauaaAUGCACGACGAu 5' hsa-miR-16
                  | | |||||||
 106:5' uuggcaggggUCCCUGCUGCUg 3' TNFSF9

     3' gaCGUCUUGACAAGGGCGACGAu 5' hsa-miR-503
          ||||::  || ||:||||||
 107:5' ugGCAGGG--GUCCCUGCUGCUg 3' TNFSF9

     3' ggguuguuguccuuuGAUGGAu 5' hsa-miR-196b
                       ||||||
  77:5' ucccugcugcuuucuCUACCUc 3' TNFSF9

     3' ggguuguuguacuuuGAUGGAu 5' hsa-miR-196a
                       ||||||
  77:5' ucccugcugcuuucuCUACCUc 3' TNFSF9

     3' uuGGUGUGUUGGA--UGAUGGAGu 5' hsa-let-7b
          || ::| :|:|   |||||||
  76:5' guCCCUGCUGCUUUCUCUACCUCa 3' TNFSF9

     3' uuguuAUGUUGAA--UGAUGGAGu 5' hsa-miR-98
             |:| :|||   |||||||
  76:5' gucccUGCUGCUUUCUCUACCUCa 3' TNFSF9

     3' uugucguguuuGAUGAUGGAGu 5' hsa-let-7i
                   || |||||||
  79:5' ccugcugcuuuCU-CUACCUCa 3' TNFSF9

     3' uuGAC-AUGUUUGAUGAUGGAGu 5' hsa-let-7g
          ||| |:|   || |||||||
  78:5' ccCUGCUGCUUUCU-CUACCUCa 3' TNFSF9

     3' uuGAU-AUGUUGGAUGAUGGAGu 5' hsa-let-7a
          ||: |:|  :|| |||||||
  78:5' ccCUGCUGCUUUCU-CUACCUCa 3' TNFSF9

     3' uuGAU-AUGUUGGAGGAUGGAGu 5' hsa-let-7e
          ||: |:|  :|| |||||||
  78:5' ccCUGCUGCUUUCU-CUACCUCa 3' TNFSF9

     3' uuGAU-AUGUUAGAUGAUGGAGu 5' hsa-let-7f
          ||: |:|  ||| |||||||
  78:5' ccCUGCUGCUUUCU-CUACCUCa 3' TNFSF9

     3' uuGAU-ACGUUGGAUGAUGGAGa 5' hsa-let-7d
          ||: |||  :|| |||||||
  78:5' ccCUGCUGCUUUCU-CUACCUCa 3' TNFSF9

     3' guguagagugacaucGGAGUUu 5' hsa-miR-1304
                       ||||||
  80:5' cugcugcuuucucuaCCUCAAg 3' TNFSF9
```

ENTPD7

Chr10: 99,704,829-99,704,866

```
     3' cgucuugaaucgguGACACUu 5' hsa-miR-27b
                      ||||||
 178:5' agcuauaauuuaauCUGUGAg 3' ENTPD7

     3' cgccuugaaucgguGACACUu 5' hsa-miR-27a
                      ||||||
 178:5' agcuauaauuuaauCUGUGAg 3' ENTPD7
```

Supplemental Figure S5D. Predicted candidates sites and complementarity for miRNA engagement with 22 representative genes. Candidate binding sites outlined in red are potential sites for binding to the experimentally determined (Figure 3) most prevalent 25 miRNAs in HCT116 cells.

TM2D2

Chr8: 38,989,050-38,989,124

```
     3' ccuuuagggaccguUACACUa 5' hsa-miR-23a
                      ||||||
2182:5' ucaucaguauuccuAUGUGAc 3' TM2D2

     3' ccauuagggaccguUACACUa 5' hsa-miR-23b
                      ||||||
2182:5' ucaucaguauuccuAUGUGAc 3' TM2D2

     3' ccUUUAGGGACCG--UUACACUa 5' hsa-miR-23a
          |||  ||||||  |||||||
2261:5' acAAA--CCUGGCCAAAUGUGAu 3' TM2D2

     3' ccauuagGGACCG--UUACACUa 5' hsa-miR-23b
               ||||||  |||||||
2259:5' ccacaaaCCUGGCCAAAUGUGAu 3' TM2D2
```

Chr8: 38,988,972-38,989,001

```
     3' agucaaAACGUAUCUAAACGUGu 5' hsa-miR-19a
              |||   |:  |||||||
2319:5' uaacaaUUGAUUGCCUUUGCACu 3' TM2D2

     3' agucaaAACGUACCUAAACGUGu 5' hsa-miR-19b
              |||   ||  |||||||
2319:5' uaacaaUUGAUUGCCUUUGCACu 3' TM2D2

     3' ugggauAUUCGUUAUAACGUGAu 5' hsa-miR-454
              |:| ::    |||||||
2320:5' aacaauUGAUUGCCUUUGCACUu 3' TM2D2

     3' cgAAACUGUUA--UGAU---AACGUGAc 5' hsa-miR-301a
          ||| |||||   |:|:    |||||||
2315:5' uaUUUAACAAUUGAUUGCCUUUGCACUu 3' TM2D2
```

```
     3' gauggacgugcuuguCGUGAAAc 5' hsa-miR-93
                       |||||||
2322:5' caauugauugccuuuGCACUUUa 3' TM2D2

     3' uaGACGUGAC--AGUCGUGAAAu 5' hsa-miR-106b
          :|| |:||  |: |||||||
2323:5' aaUUG-AUUGCCUUUGCACUUUa 3' TM2D2

     3' gaUGGACGUGAC--AUUCGUGAAAc 5' hsa-miR-17
          | :|| |:||  |  |||||||
2321:5' acAAUUG-AUUGCCUUUGCACUUUa 3' TM2D2

     3' gaugGACGUGAUAUUCGUGAAAu 5' hsa-miR-20a
            :||| || |   |||||||
2325:5' uugaUUGC-CUUU--GCACUUUa 3' TM2D2
```

MSMO1/SC4MOL

Chr4: 165,342,972-165,343,087

```
     3' aguugcccucacuagcACAGUAa 5' hsa-miR-425
                        ||||||
1020:5' uauaucaaauuuccauUGUCAUu 3' SC4MOL

     3' agucaaAACGUAUCUAAACGUGu 5' hsa-miR-19a
              |||| |: ||||||||
1069:5' acugacUUGC-UGUAUUUGCACu 3' SC4MOL

     3' agucaaAACGUACCUAAACGUGu 5' hsa-miR-19b
              |||| || ||||||||
1069:5' acugacUUGC-UGUAUUUGCACu 3' SC4MOL

     3' ugggauauuCGUUAU-AACGUGAu 5' hsa-miR-454
                 || :|| |||||||
1068:5' aacugacuuGCUGUAUUUGCACUu 3' SC4MOL
```

```
     3' cgaaACUGUUAUGAU---AACGUGAc 5' hsa-miR-301a
            ||||  |:||:    |||||||
1067:5' gaacUGAC-UUGCUGUAUUUGCACUu 3' SC4MOL

     3' gaUGGACGUGCUUGUCGUGAAAc 5' hsa-miR-93
          ||:|||    | : |||||||
1071:5' ugACUUGCUGUAUUUGCACUUUg 3' SC4MOL

     3' uaGACGUGACAGUCGUGAAAu 5' hsa-miR-106b
          :|||   | |: |||||||
1073:5' acUUGCUGUAUUUGCACUUUg 3' SC4MOL

     3' gaUGGACGUGACAUUCGUGAAAc 5' hsa-miR-17
          ||:|||   | |  |||||||
1071:5' ugACUUGCUGUAUUUGCACUUUg 3' SC4MOL
```

```
     3' gaUGGACGUGAUAUUCGUGAAAu 5' hsa-miR-20a
          ||:|||  |||  |||||||
1071:5' ugACUUGCUGUAUUUGCACUUUg 3' SC4MOL

     3' ccuuuagggaccgUUACACUa 5' hsa-miR-23a
                     |||||||
1094:5' agcucuugaaauaAAUGUGAu 3' SC4MOL

     3' ccAUUAGGGACC----GUUACACUa 5' hsa-miR-23b
          |:| |:|| |      |||||||
1090:5' uuUGAGCUCUUGAAAUAAAUGUGAu 3' SC4MOL
```

TUBB

Chr6: 30,724,896-30,724,970

```
    3' gaUGGACGUGCUUGUCGUGAAAc 5' hsa-miR-93
         | | |:|: | |  ||||||
537:5' ucAGCAGUAUUAUCUCCACUUUc 3' TUBB

    3' uaGACGUGACAGUCGUGAAAu 5' hsa-miR-106b
         | |:|:| ||  ||||||
539:5' agCAGUAUUAUCUCCACUUUc 3' TUBB

    3' gaUGGACGUGACAUUCGUGAAAc 5' hsa-miR-17
         | | |:|:| |   ||||||
537:5' ucAGCAGUAUUAUCUCCACUUUc 3' TUBB

    3' gaUGGACGUGAUAUUCGUGAAAu 5' hsa-miR-20a
         | | |:|:|||   ||||||
537:5' ucAGCAGUAUUAUCUCCACUUUc 3' TUBB
```

Chr6: 30,724,970-30,725,063

```
    3' ggguuguuguacuuuGAUGGAu 5' hsa-miR-196a
                      ||||||
616:5' cuucccuuuccaacuCUACCUc 3' TUBB

    3' ggguuguuguccuuuGAUGGAu 5' hsa-miR-196b
                      ||||||
616:5' cuucccuuuccaacuCUACCUc 3' TUBB

    3' uuGAUAUGUUGGAGGAUGGAGu 5' hsa-let-7e
         || | ||||   :|||||||
619:5' ccCUUUCCAAC--UCUACCUCc 3' TUBB

    3' uugucguGUUUGAUGAUGGAGu 5' hsa-let-7i
              | |||| |||||||
618:5' ucccuuuCCAACU-CUACCUCc 3' TUBB
```

```
    3' uuGGUGUGUUGGAUGAUGGAGu 5' hsa-let-7b
         || : | | || |||||||
618:5' ucCCUUUCCAACU-CUACCUCc 3' TUBB

    3' uuGAUAUGUUAGAUGAUGGAGu 5' hsa-let-7f
         || | ||| || |||||||
619:5' ccCUUUCCAA-CU-CUACCUCc 3' TUBB

    3' uuGACAUGUUUGAUGAUGGAGu 5' hsa-let-7g
         || | | |||| |||||||
619:5' ccCU-UUCCAACU-CUACCUCc 3' TUBB

    3' uuGAUAUGUUGGAUGAUGGAGu 5' hsa-let-7a
         || | ||||:  |||||||
619:5' ccCUUUCCAACU--CUACCUCc 3' TUBB

    3' uuGAUACGUUGGAUGAUGGAGa 5' hsa-let-7d
         || | ||||:  |||||||
619:5' ccCUUUCCAACU--CUACCUCc 3' TUBB

    3' uuguuAUGUUGAAUGAUGGAGu 5' hsa-miR-98
            | |||||  |||||||
619:5' cccuuUCCAACU--CUACCUCc 3' TUBB
```

GPRC5A

Chr12:12,915,880-12,915,922

```
     3' uguuucaagacaucACGUGACu 5' hsa-miR-148a
                      |||||||
3337:5' gggcaggccagcccUGCACUGa 3' GPRC5A

     3' uguuucaagacacuACGUGACu 5' hsa-miR-148b
                      |||||||
3337:5' gggcaggccagcccUGCACUGa 3' GPRC5A
```

```
     3' ucgauaCGGucguAGAACGGa 5' hsa-miR-31
              |||    |||||||
3353:5' cugaacGCCugu-UCUUGCCa 3' GPRC5A

     3' gcgguuauaaaugcACGACGAu 5' hsa-miR-16
                      |||||||
3374:5' agguggcagaagguUGCUGCUc 3' GPRC5A
```

```
     3' guguuugguaauacACGACGAu 5' hsa-miR-15a
                      |||||||
3374:5' agguggcagaagguUGCUGCUc 3' GPRC5A

     3' acauuugguacuacACGACGAu 5' hsa-miR-15b
                      |||||||
3374:5' agguggcagaagguUGCUGCUc 3' GPRC5A

     3' aaguuuuguacuuaACGACGAc 5' hsa-miR-424
                      |||||||
3374:5' agguggcagaagguUGCUGCUc 3' GPRC5A
```

TSPAN13

Chr7:16,783,988-16,784,002

```
    3' ggUAGAAAUG--GUCUGUCACAAu 5' hsa-miR-141
         ||: ||::  :| | ||||||
488:5' agAUUGUUGUGGUAAAAAGUGUUa 3' TSPAN13

    3' ugUAGCAAUG--GUCUGUCACAAu 5' hsa-miR-200a
         ||:|||::  :| | ||||||
488:5' agAUUGUUGUGGUAAAAAGUGUUa 3' TSPAN13
```

Supplemental Figure S5D. Predicted candidates sites and complementarity for miRNA engagement with 22 representative genes. Candidate binding sites outlined in red are potential sites for binding to the experimentally determined (Figure 3) most prevalent 25 miRNAs in HCT116 cells.

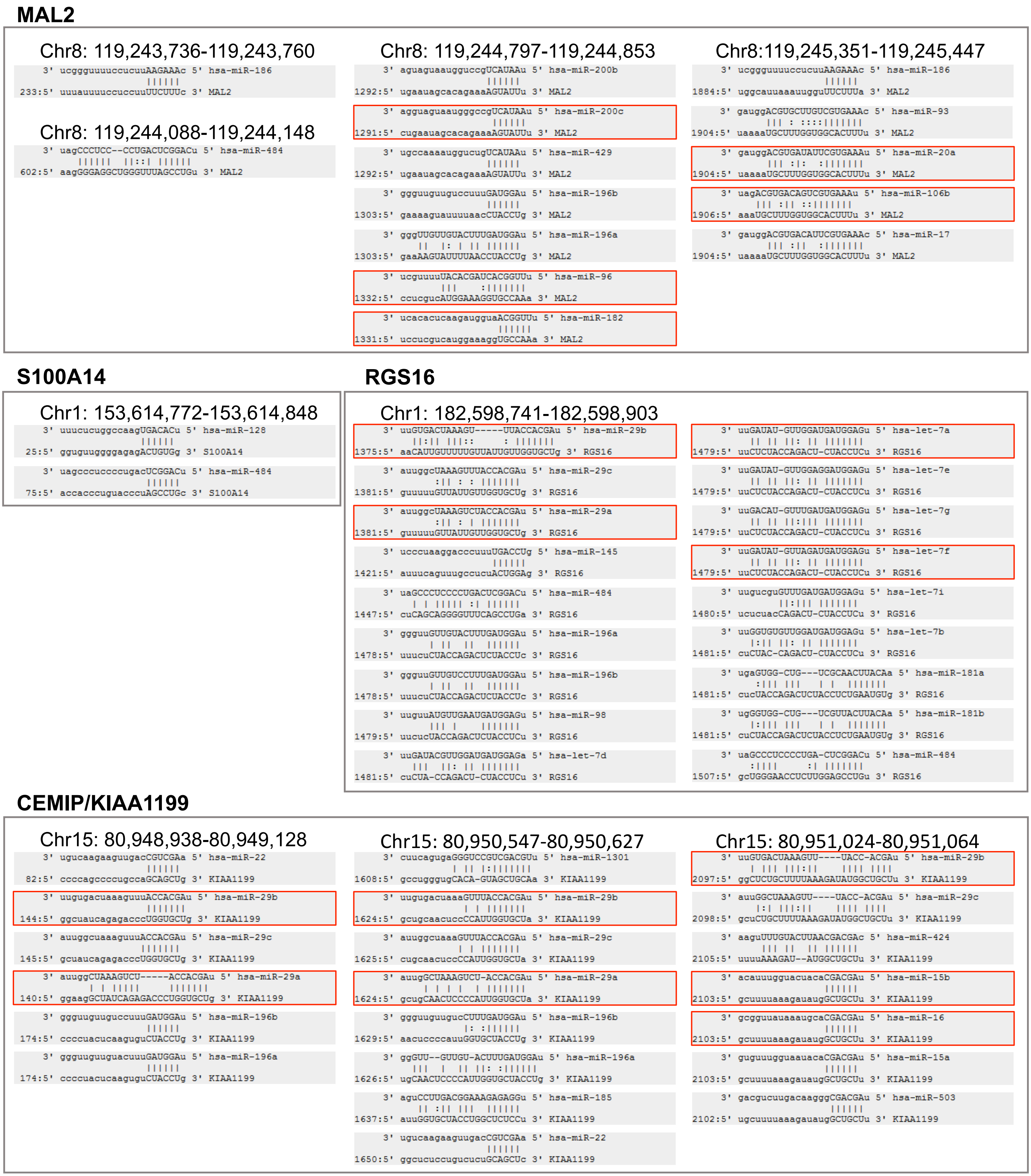

Supplemental Figure S5D. Predicted candidates sites and complementarity for miRNA engagement with 22 representative genes. Candidate binding sites outlined in red are potential sites for binding to the experimentally determined (Figure 3) most prevalent 25 miRNAs in HCT116 cells.

GALNT3

Chr2:165,748,518-165,748,643

```
    3' uuAGUCAGAGUAACGAAAUAUu 5' hsa-miR-340
         ||   :|:||  ||||||:
123:5' uuUCUUAUUUAUAUCUUUAUGu 3' GALNT3

    3' cuuugggucgucuguUACAUCGa 5' hsa-miR-221
                      |||||||
126:5' cuuauuuuauaucuuuAUGUAGCa 3' GALNT3

    3' ugggucAUCGGUCUACAUCGa 5' hsa-miR-222
             || |:  ||||||
128:5' uauuuaUAUCUUUAUGUAGCa 3' GALNT3

    3' aguaUGUCGAUC-UAUUGGUUUCu 5' hsa-miR-9
           |:| |||| |  |||||||
184:5' cacaAUAACUAGUAAUACCAAAGa 3' GALNT3

    3' gaaggucagUUC-CUACAAAUGu 5' hsa-miR-30e
                ||| |||||||||
226:5' auguaggggAAGAGAGUGUUUACa 3' GALNT3

    3' gaaggucagcCCCUACAAAUGu 5' hsa-miR-30d
                 | |||||||||
227:5' uguaggggaaGAGAUGUUUACa 3' GALNT3

    3' ucgACUCACAUC-CUACAAAUGu 5' hsa-miR-30b
          | :| | || |||||||||
226:5' augUAGGGGAAGAGAUGUUUACa 3' GALNT3

    3' cgAC-UCUCACAUC-CUACAAAUGu 5' hsa-miR-30c
         || ||:| | || |||||||||
225:5' gaUGUAGGG-GAAGAGAUGUUUACa 3' GALNT3

    3' aguaguaaugguccGUCAUAau 5' hsa-miR-200b
                     ||||||
233:5' ggaagagaguguuuaCAGUAUga 3' GALNT3

    3' agguaguaaugggccGUCAUAau 5' hsa-miR-200c
                      ||||||
232:5' gggaagagaguguuuaCAGUAUga 3' GALNT3

    3' ugccaaaaugguc UGUCAUAau 5' hsa-miR-429
                    |||||||
233:5' ggaagagaguguuuACAGUAUga 3' GALNT3
```

MYC

Chr8: 127,741,018-127,741,130

```
    3' cgUUACGUUGUCG-UUACGUg 5' hsa-miR-33b
         ||||||  | | ||||||
66:5' caAAUGCAUGAUCAAAUGCAa 3' MYC

    3' acgUUACGU-UGAUGUUACGUg 5' hsa-miR-33a
          |||||| : |  ||||||
65:5' ucaAAUGCAUGAUCAAAUGCAa 3' MYC

    3' aguguauccuuacuuuUCGGUAu 5' hsa-miR-135b
                       ||||||
109:5' ugagacugaaagauuuAGCCAUa 3' MYC

    3' ugUUGGUCGAUU-C-UGUGACGGu 5' hsa-miR-34a
         |:|||  ||| | | ||||||
123:5' uuAGCCA--UAAUGUAAACUGCCu 3' MYC

    3' uuGGUGUGUUGGAUGAUGGAGu 5' hsa-let-7b
         |||:|  ::  |||:||||
125:5' agCCAUAAUGUAAACUGCCUCa 3' MYC

    3' uuGAUAUGUUGGAUGAUGGAGu 5' hsa-let-7a
         | |||  ::  |||:||||
125:5' agCCAUAAUGUAAACUGCCUCa 3' MYC

    3' uuGAUAUGUUGGAGGAUGGAGu 5' hsa-let-7e
         | |||  ::   ||:||||
125:5' agCCAUAAUGUAAACUGCCUCa 3' MYC

    3' uuGAUAUGUUAGAUGAUGGAGu 5' hsa-let-7f
         | |||  :|  |||:||||
125:5' agCCAUAAUGUAAACUGCCUCa 3' MYC

    3' uuGUC-GUGUUUGA--UGAUGGAGu 5' hsa-let-7i
         :|| ||:||  |   |||:||||
122:5' uuUAGCCAUAAUGUAAACUGCCUCa 3' MYC

    3' uuGUUAUGUUGAAUGAUGGAGu 5' hsa-miR-98
         :|||::||   |||:||||
128:5' caUAAUGUAA---ACUGCCUCa 3' MYC

    3' uugacaUGUUUGA--UGAUGGAGu 5' hsa-let-7g
             |:||  |   |||:||||
123:5' uuagccAUAAUGUAAACUGCCUCa 3' MYC

    3' uugaUACGUUGGAUGAUGGAGa 5' hsa-let-7d
           |||:||   |||:||||
128:5' cauaAUGUAA---ACUGCCUCa 3' MYC

    3' guguaGAGUGACAU----CGGAGUUu 5' hsa-miR-1304
            | :| ||||     |||||||
123:5' uuagcCAUAAUGUAAACUGCCUCAAa 3' MYC
```

ABCC2

Chr10: 99,851,744-99,851,887

```
    3' ucggauaGGACCUAAUGAACUu 5' hsa-miR-26a
              || ||  |||||||
160:5' aaugucaCCAGG--UACUUGAg 3' ABCC2

    3' ggguuguguacuuuGAUGGAu 5' hsa-miR-196a
                     ||||||
180:5' aaaccccucgauuguCUACCUc 3' ABCC2

    3' ggguuguguccuuuGAUGGAu 5' hsa-miR-196b
                     |||||
180:5' aaaccccucgauuguCUACCUc 3' ABCC2

    3' uugauauGUUGGAUGAUGGAGu 5' hsa-let-7a
              |:|::  |||||||
181:5' aaccccuCGAUUGUCUACCUCg 3' ABCC2

    3' uugauacGUUGGAUGAUGGAGa 5' hsa-let-7d
              |:|::  |||||||
181:5' aaccccuCGAUUGUCUACCUCg 3' ABCC2

    3' uugauauGUUGGAGGAUGGAGu 5' hsa-let-7e
              |:|:: :|||||||
181:5' aaccccuCGAUUGUCUACCUCg 3' ABCC2

    3' uugacauguUUGAU-GAUGGAGu 5' hsa-let-7g
                :|:|: |||||||
180:5' aaaccccucGAUUGUCUACCUCg 3' ABCC2

    3' uuguuauGUUGAAUGAUGGAGu 5' hsa-miR-98
              |:|:|  |||||||
181:5' aaccccuCGAUUGUCUACCUCg 3' ABCC2

    3' uuGGUGUGUUGGAUGAUGGAGu 5' hsa-let-7b
         || |  || |:|::  |||||||
181:5' aaCCCCUCGAUUGUCUACCUCg 3' ABCC2

    3' uugauauGUUAGAUGAUGGAGu 5' hsa-let-7f
              |:||:  |||||||
181:5' aaccccuCGAUUGUCUACCUCg 3' ABCC2

    3' uugucGUGUUUGAUGAUGGAGu 5' hsa-let-7i
            | |:| :  |||||||
181:5' aacccCUCGAUUGUCUACCUCg 3' ABCC2
```

DDIT

Chr10:72,275,251-72,275,318

```
    3' uguagcaauggucUGUCACAAu 5' hsa-miR-200a
                    |:||||||
127:5' uggaggugggggaAUAGUGUUu 3' DDIT4

    3' gguagaaaugguc UGUCACAAu 5' hsa-miR-141
                    |:||||||
127:5' uggaggugggggaAUAGUGUUu 3' DDIT4
```

Chr10:72,275,845-72,275,946

```
    3' ucacuuaaagauggUCACGGUAu 5' hsa-miR-183
                     ||||||||
700:5' ccuccccgggaggAGUGCCAUc 3' DDIT4

    3' gaAGGUCAGCCCCU-ACAAAUGu 5' hsa-miR-30d
         |||| ||   | | |||||||
727:5' cuUCCA-UCUAGAACUGUUUACa 3' DDIT4

    3' ucgacucACAUCCU-ACAAAUGu 5' hsa-miR-30b
              | ||| | |||||||
726:5' ucucccaUCUAGAACUGUUUACa 3' DDIT4

    3' cgacucucACAUCCU-ACAAAUGu 5' hsa-miR-30c
               | ||| | |||||||
725:5' gucuuccaUCUAGAACUGUUUACa 3' DDIT4

    3' gaAGGUCAGUUCCU-ACAAAUGu 5' hsa-miR-30e
         |||| || || | |||||||
727:5' cuUCCA-UCUAGAACUGUUUACa 3' DDIT4

    3' uuUCUCUGGCCAAGUGACAcu 5' hsa-miR-128
         ||| ||     |||||||
755:5' uaAGAUAC----UCACUGUuc 3' DDIT4
```

Chr10:72,275,346-72,275,651

```
    3' aagucaauaGUGUCAUGACAu 5' hsa-miR-101
                | |||||||||
195:5' cacauacccCUCAGUACUGUa 3' DDIT4

    3' ugggucaucggucuACAUCGa 5' hsa-miR-222
                     ||||||
198:5' auaccccucaguacUGUAGCa 3' DDIT4

    3' cuuUGGGUCGUCUGUUACAUCGa 5' hsa-miR-221
          ||||  |||  :| ||||||
197:5' cauACCCCUCAG-UACUGUAGCa 3' DDIT4

    3' ugGGUC-AUCGGU-C-UACAUCGa 5' hsa-miR-222
         |||| |:| :: | :||||||
248:5' uuCCAGCUGGAUGUGUGUGUAGCa 3' DDIT4

    3' cuuuGGGUCG-UCUGU---UACAUCGa 5' hsa-miR-221
          :||||| :||::    :||||||
245:5' ggcuUCCAGCUGGAUGUGUGUGUAGCa 3' DDIT4

    3' ugucaagaaguugacCGUCGAa 5' hsa-miR-22
                      ||||||
310:5' ugugacauccagagaGCAGCUg 3' DDIT4

    3' gcgguuauaaaugcaCGACGAu 5' hsa-miR-16
                      ||||||
318:5' ccagagagcagcuggGCUGCUc 3' DDIT4

    3' gugUUUGGUAAUACACGACGAu 5' hsa-miR-15a
          |:| ||    || ||||||
319:5' cagAGAGCA-GCUGGGCUGCUc 3' DDIT4

    3' acaUUUGGUACUACACGACGAu 5' hsa-miR-15b
          |:| || | | || ||||||
319:5' cagAGAGCA-GCUGGGCUGCUc 3' DDIT4

    3' aaguuuuguacuuaaCGACGAc 5' hsa-miR-424
                      ||||||
318:5' ccagagagcagcuggGCUGCUc 3' DDIT4

    3' gacgUCUUGACAAGGGCGACGAu 5' hsa-miR-503
           |||:| | |    ||||||
318:5' ccagAGAGCAGCU-GGGCUGCUc 3' DDIT4

    3' guguuuaagccuagauGUCCCAu 5' hsa-miR-10a
                       ||||||
340:5' ccgccccagcccggccCAGGGUg 3' DDIT4

    3' guguuuaagccaagauGUCCCAu 5' hsa-miR-10b
                       ||||||
340:5' ccgccccagcccggccCAGGGUg 3' DDIT4
```

PRSS8

Chr16:31,131,462-31,131,799

```
    3' cuuugggucgucuguuACAUCGa 5' hsa-miR-221
                      ||||||
189:5' caggaguuuuacugccUGUAGCa 3' PRSS8

    3' ugggucaucggucuACAUCGa 5' hsa-miR-222
                     ||||||
191:5' ggaguuuuacugccUGUAGCa 3' PRSS8

    3' gugCUGGCUGCGGU--GCGGCUCa 5' hsa-miR-1307
          |||| :| |||    ||||||
242:5' augGACCAGC-CCAUUGGCCGAGc 3' PRSS8

    3' gacaaggacgacuugACUCGGu 5' hsa-miR-24
                      ||||||
285:5' ccuuggcuaugaaaaUGAGCCc 3' PRSS8

    3' aguccuugacggaaaGAGAGGu 5' hsa-miR-185
                      ||||||
362:5' gcacaucucucugccCUCUCCc 3' PRSS8

    3' gauAGACGUGAUCUACGUGGAAu 5' hsa-miR-18a
          |||| |||:|:   ||||||
386:5' uguUCUGGGCUGGGGCCACCUUu 3' PRSS8

    3' ugucaagaaguugacCGUCGAa 5' hsa-miR-22
                      ||||||
396:5' uggggccaccuuuguGCAGCUu 3' PRSS8

    3' ucgaUACGGUCGUAGAACGGa 5' hsa-miR-31
           | |||   ||||||||
426:5' ggaaAGGCCCCAAUCUUGCCc 3' PRSS8

    3' gacaAGGACGACUUGACUCGGu 5' hsa-miR-24
           |||  |   |:||||||||
481:5' ggacUCC-GGAGGACUGAGCCc 3' PRSS8

    3' ugugugggugcgcucGGCCUUu 5' hsa-miR-1180
                      ||||||
490:5' aggacugagcccccaCCGGAAc 3' PRSS8
```

Supplemental Figure S5D. Predicted candidates sites and complementarity for miRNA engagement with 22 representative genes. Candidate binding sites outlined in red are potential sites for binding to the experimentally determined (Figure 3) most prevalent 25 miRNAs in HCT116 cells.

TMEM184A

Chr7:1,546,895-1,546,926

```
   3' guguuuaagCCUAGAUGUCCCAu 5' hsa-miR-10a
               |||   |||||||
43:5' ggccucuggGGAAGAACAGGGUc 3' TMEM184A
```

```
   3' guguuuaagccaagaUGUCCCAu 5' hsa-miR-10b
                     |||||||
43:5' ggccucuggggaagaACAGGGUc 3' TMEM184A
```

EFNA1

Chr1:155,134,333-155,134,399

```
    3' aguaugucgaucuaUUGGUUUCu 5' hsa-miR-9
                     |:||||||
257:5' cauuccugccuuuaAGCCAAAGa 3' EFNA1
```

Chr1:155,134,738-155,134,783

```
    3' aaGUCAAUAGUGUCAU-GACAu 5' hsa-miR-101
         ||  |:| |:|||| ||||
677:5' ccCACGUGU-AUAGUAUCUGUa 3' EFNA1
```

Chr1:155,134,513-155,134,694

```
    3' cuuugggucgucuguuACAUCGa 5' hsa-miR-221
                       ||||||
510:5' cugccacagagaaguuUGUAGCc 3' EFNA1
```

```
    3' ugggucaucggucuACAUCGa 5' hsa-miR-222
                     ||||||
512:5' gccacagagaaguuUGUAGCc 3' EFNA1
```

```
    3' cccUCACUUCUGUGCCUCGGUCu 5' hsa-miR-149
          || |||| : :| ||||||
514:5' cacAGAGAAG-UUUGUAGCCAGg 3' EFNA1
```

```
    3' agUCCUUGACGGAAAGAGAGGu 5' hsa-miR-185
         ||| |||| | ||||||||
531:5' ccAGGUACUG-CAUUCUCUCCc 3' EFNA1
```

```
    3' ucacacucaagaugguaACGGUUu 5' hsa-miR-182
                        ||||||
579:5' ugccagcagggggcugUGCCAAc 3' EFNA1
```

```
    3' ucGUU-UUUACACGAUCACGGUUu 5' hsa-miR-96
         ||: |:: | ||| |||||||
580:5' gcCAGCAGGGGGCU-GUGCCAAc 3' EFNA1
```

```
    3' cuUUGGGU-CGUCUGUUACAUCGa 5' hsa-miR-221
         ||||::  |  | |:||||||
598:5' ccAACCUGUUCUUAGAGUGUAGCu 3' EFNA1
```

```
    3' ugGGUCAUCGGUC-UACAUCGa 5' hsa-miR-222
         || ||  ::|| :||||||
600:5' aaCCUGUUCUUAGAGUGUAGCu 3' EFNA1
```

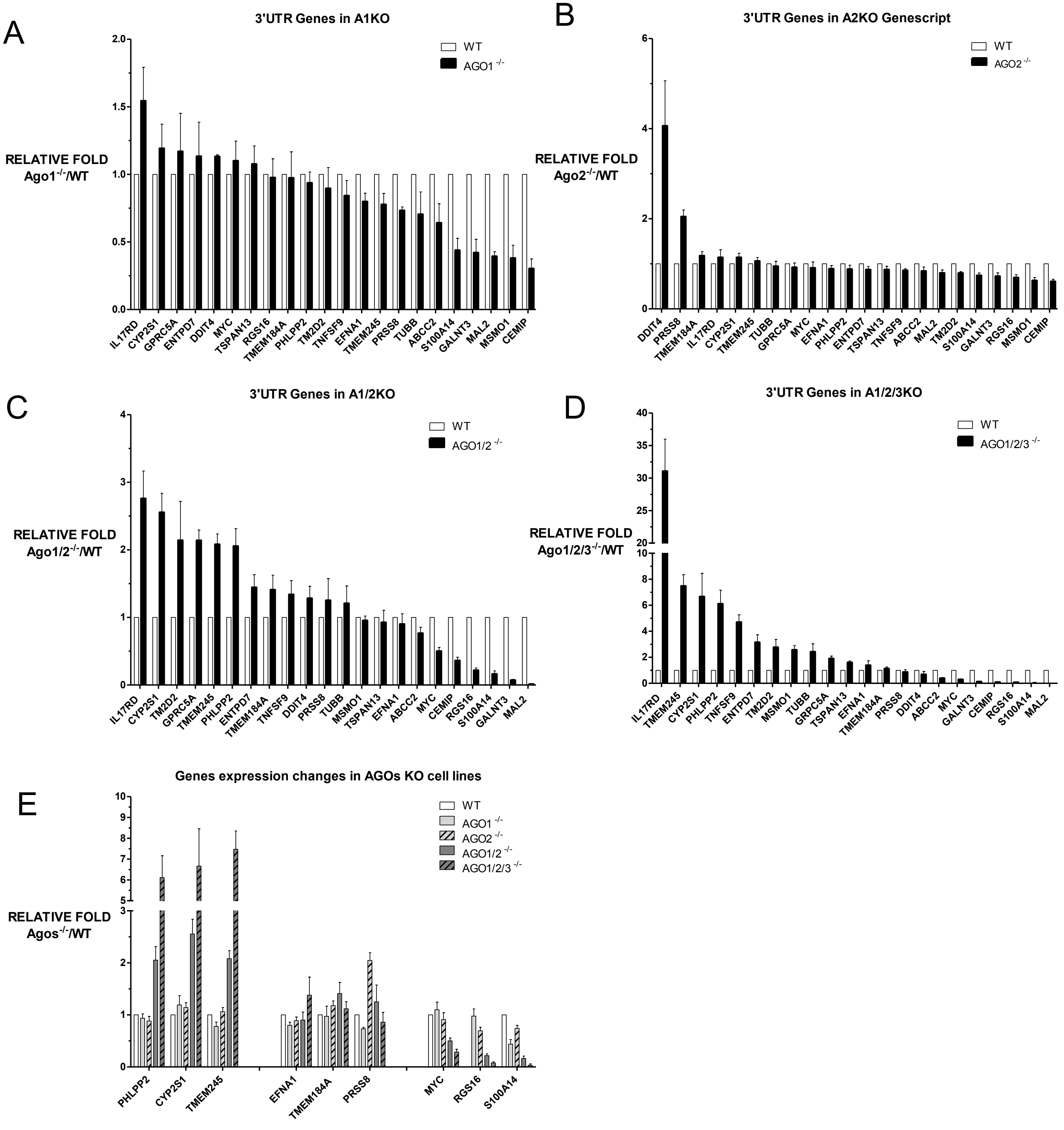

Supplemental Figure S5E (Related to Figure 7). Expression level change of genes with AGO2 binding clusters located at 3'UTR measured by QPCR in AGO1$^{-/-}$ cell line (A), AGO2$^{-/-}$ cell line (B), AGO1/2$^{-/-}$ cell line (C) and AGO1/2/3$^{-/-}$ cell line (D). (E) Representative gene expression changes in AGOs knockout cell lines.

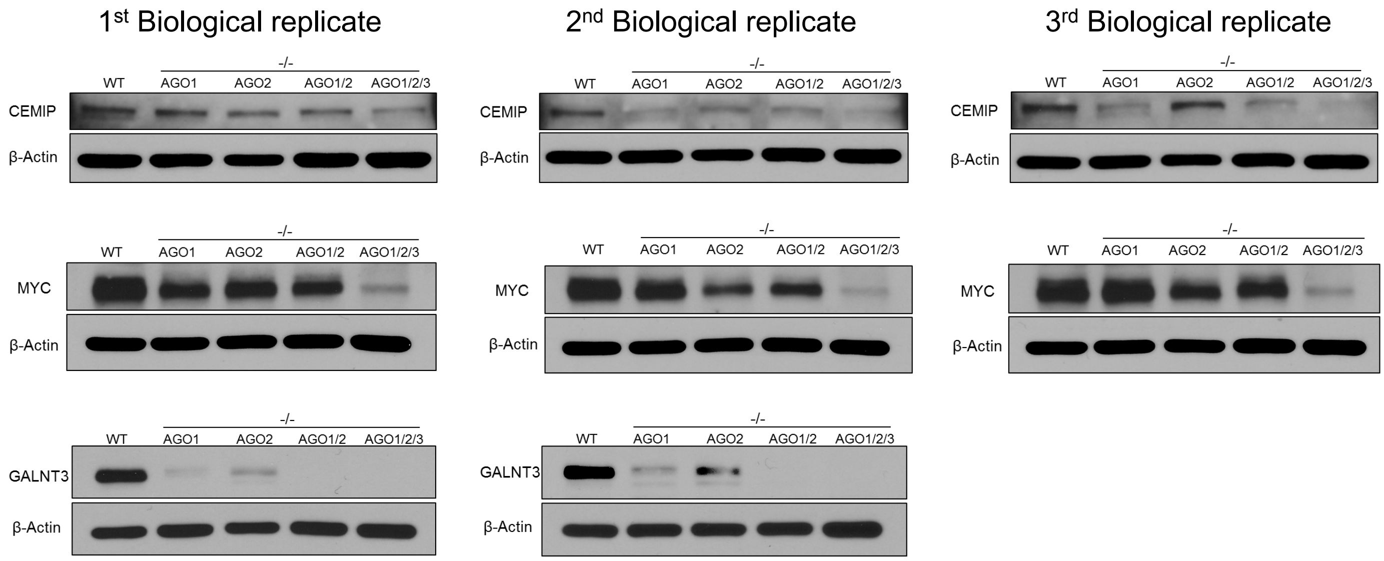

Figure S5F (Related to Figure 7). Biological replicates of Western-blots.

A

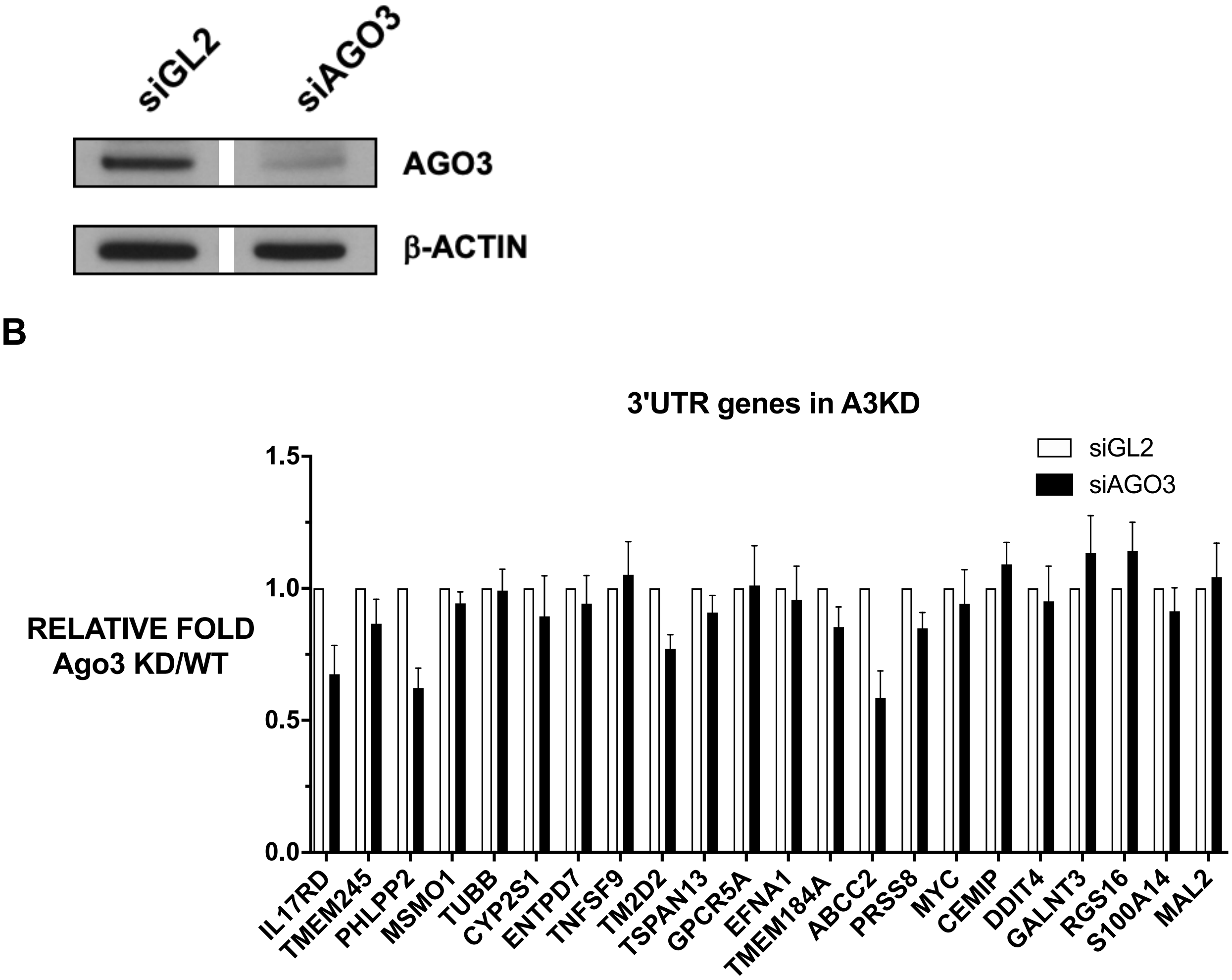

Supplemental Figure S6 . Effect of AGO3 knock down Expression level change of genes with AGO2 binding clusters located at 3'UTR by AGO3-siRNA. (A) Western analysis of AGO3 expression in siAGO3 transfection cell. **(B)** Expression level change of 22 genes with AGO2 binding cluster in 3'UTR. siGL2 is a noncomplementary control duplex RNA. The knockdown was achieved using a duplex RNA complementary to AGO3 mRNA 5'-GCAUCAUUAUGCAAUAUGAUU-3'/5'-pUCAUAUUGCAUAAUGAUGCUU-3'. The RNA was delivered into HCT116 cells using cationic lipid Lipofectamine RNAiMax or Lipofectamine 3000.

(A)

(B)

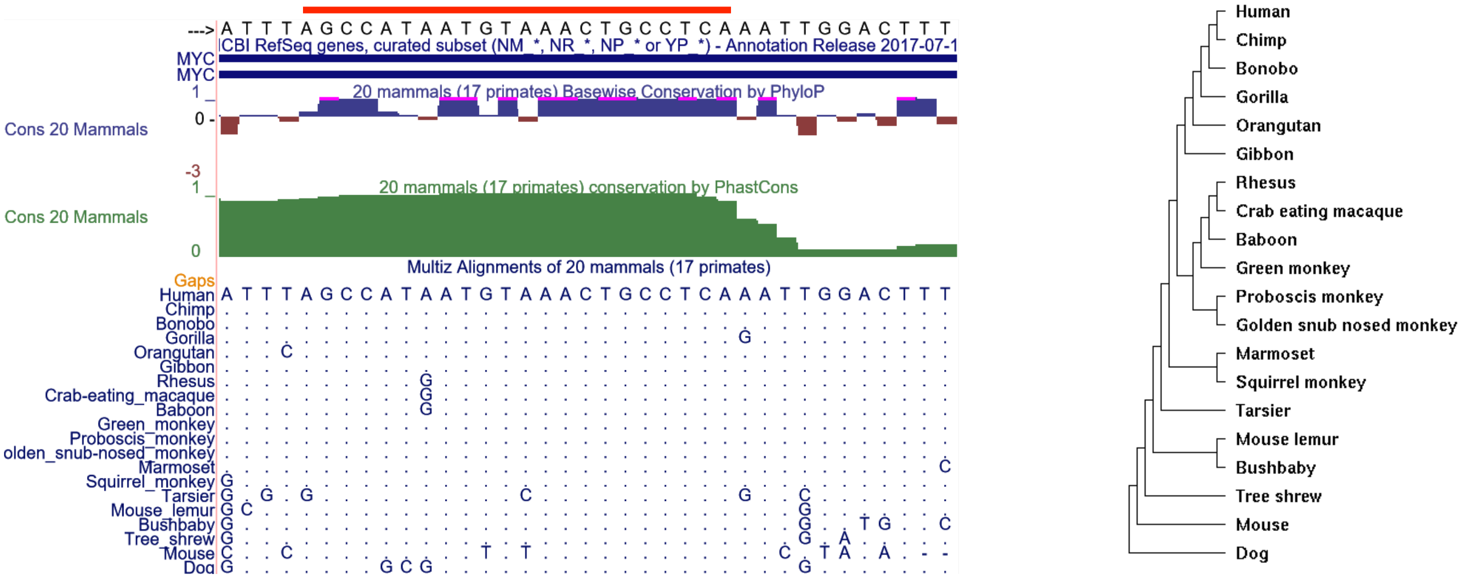

Supplementary Figure S7. The sequence conservation analysis of a let-7 binding site within *MYC* 3'-UTR. (**A**) The binding site sequence alignments for 20 mammals. The PhyloP and PhastCons scores are also shown in the top part. (**B**) Phylogenetic tree model generated based on the sequence alignments for 20 mammals.

Supplementary Table 1. Primer sequences for validated genes.

Gene		Primer sequence 5′-3′
ABCC2	Set 1	For: TGGATCTAGAGACAGACAACCT
		Rev: CTCTATAATCTTCCCGTTGTCTAGG
	Set 2	For: GGACAACCACTGTCCTGAAT
		Rev:TGCAGTATGAGTCAGTCCATTT
CEMIP	Set 1	For: GCACATCCTGATTGACAACG
		Rev: ACCAACCCCAATGTACTTCAG
	Set 2	For: ATCAGGAGACCTGGGTTGTG
		Rev: ACCAACCCCAATGTACTTCAG
CYP2S1	Set 1	For: TGTATTCAGGGCTCATGCG
		Rev: TCAAAAGTCCCTTCCAGCATC
	Set 2	For: GCCTCCCTTACACCGAC
		Rev: CTTCCTGAACCGTCCATCTG
DDIT4	Set 1	For: AGCAACAGTGGCTTCGG
		Rev: GGCACACAAGTGTTCATCCT
	Set 2	For: GTGTATCTTACTGGTCTGAAGGG
		Rev: GATAGCTGCCTACAACAGGTC
EFNA1	Set 1	For: AGACCCATAGGAGACCCG
		Rev: CGGAACTTGGGATTTGAACTG
	Set 2	For: TCTGGAACAGTTCAAATCCCA
		Rev: TGATCTTCATAGTGCGGACAG
ENTPD7	Set 1	For: GGGTTTATGCATGGATTGGAATC
		Rev: AGGACAGAGGTTGAGGTAGG
	Set 2	For: CCTATGACTACCCAAACCTGC
		Rev: CGCAGAAGGTATAGGAAGATGG
GALNT3	Set 1	For: GGAGTCGCTTCCTGATCATG
		Rev: CCTCCCCAGATTTCCATTTCT
	Set 2	For: GGTGGATGATGCTAGTGTAGATG
		Rev: GTTTCAGCTGTTGCGACTG
GPRC5A	Set 1	For: GGGACACGCTCTATGCC
		Rev: TAGCTGCCCTCTTTCTTTACTT
	Set 2	For: TTCCTCTCCTTTCCCTTTGC
		Rev: ATCCCTTTCCCATGGCTTAC
IL17RD	Set 1	For: CTTTAGAACCCGAGCCTGTG
		Rev: CGTCTCTGTAGTTTGCTCCTG
	Set 2	For: CATCTATGACTCGTCTGTGCC
		Rev: CCAAGATCTGCTTTGCATGAC
MAL2	Set 1	For: ATACAACCATAACCGGGCAG
		Rev: TTTCTAAGGAGTGTTACGGTCG
	Set 2	For: CTTCGTCTGCCTGGAGATTC
		Rev: AGAGGAACATGCCCAGAAAG
MSMO1	Set 1	For: ATCCCTTATAGTTCATGAAGCCC
		Rev: GGTTCCACAAATCAAAGGCAG
	Set 2	For: TGCACATCCTTTGGAGACTC
		Rev: CCGAGAACCAGCATAGAAAGG
MYC	Set 1	For: TCTTCCCCTACCCTCTCAAC
		Rev: ACCAACCCCAATGTACTTCAG
	Set 2	For: TGAACAGCTACGGAACTCTTG
		Rev: ACCAACCCCAATGTACTTCAG
PELI2	Set 1	For: ATAAGGAGCCAGTGAAATACGG
		Rev: CTGATAGCCTTGGATGCCTG
	Set 2	For: CTAATGGCGTCCTGGTGATG
		Rev: TGTGCTTCTGAGTTGGAGTATG
PHLPP2	Set 1	For: CACATTTTGCCTCTGGTTGG
		Rev: GACATGATAGGTCTGAGCTTGG
	Set 2	For: GGAGGCTCAAAGGGTGAAG
		Rev: TGCTAATGGGTCTTGTACGTG
PRSS8	Set 1	For: GGCCATTCTGCTCTATCTTGG
		Rev: ACACATGGACGCCTTCATAG
	Set 2	For: CCTATGAAGGCGTCCATGTG
		Rev: CTCGGAGTAGGAGTCTAGCTG
RGS16	Set 1	For: TGCTAAACCCAACCGCAG
		Rev: GAAAGATCCCCAGACGTGTC
	Set 2	For: GAAGATCCGATCAGCTACCAAG
		Rev: AGTCCTTCTCCATCAGGGTAC
S100A14	Set 1	For: ACAGATCATGAGCCATCAGC
		Rev: CCCTCTCCACATCACTGAATTC
	Set 2	For: AACTTTCACCAGTACTCCGTG
		Rev: GGCAATTTTCTCTTCCAGGC
TM2D2	Set 1	For: CAGTGGATCATGTTGGAAATGC
		Rev: AGGACTGGCACACTCAATTC
	Set 2	For: AATTGAGTGTGCCAGTCCTAG
		Rev: CATCAGCCCTCCAGTAATTAGC
TMEM184A	Set 1	For: CCACAGGAGCAACGTTACATC
		Rev: GGAAACACAGGCTCAGGAAG
	Set 2	For: GTCATCTTCCTGTCGTTCTGG
		Rev: CTTCTTCTCTGCGTACACCTG
TMEM245	Set 1	For: TCCTGTACGGCCTCTACTG
		Rev: GAAACAGTCAACACATAGCCAAC
	Set 2	For: CTAGTGAGTCCCACGAATTCAG
		Rev: GCAGAGGAAACCACATCAACC
TNFSF9	Set 1	For: CTGGTGGCCCAAAATGTTC
		Rev: CTCCTTCGTGTCCTCTTTGTAG
	Set 2	For: GAGGGTCCCGAGCTTTC
		Rev: GGTCACTGTACCAGCTCAG
TUBB	Set 1	For: CAAGTTCTGGGAGGTGATCAG
		Rev: GATGGCACGAGGAACATATTTG
	Set 2	For: GGCCAGATCTTTAGACCAGAC
		Rev: CCTTCCGTACCACATCCAG
HPRT1		For: AGTTCTGTGGCCATCTGCTTAGTAG
		Rev: AAACAACAATCCGCCCAAAGG
snRNP200		For: CGAGAAGTGGGACATCATCAC
		Rev: ACCTCTGTCATCGTGGAGAA

Supplementary Table 2. Oligonucleotides used in eCLIP library preparation

**Oligonucleotides for eCLIP**		
**Oligonucleotide name**	**Description**	**Sequence (5' to 3')**
RNA_A01	Library preparation	/5phos/rArUrUrGrCrUrUrArGrArUrCrGrGrArArGrArGrCrGrUrCrGrUrGrUrArG/3SpC3/
RNA_B06	Library preparation	/5phos/rArCrArArGrCrCrArGrArUrCrGrGrArArGrArGrCrGrUrCrGrUrGrUrArG/3SpC3/
RNA_C01	Library preparation	/5phos/rArArCrUrUrGrUrArGrArUrCrGrGrArArGrArGrCrGrUrCrGrUrGrUrArG/3SpC3/
RNA_D08	Library preparation	/5phos/rArGrGrArCrCrArArGrArUrCrGrGrArArGrArGrCrGrUrCrGrUrGrUrArG/3SpC3/
RNA_X1A	Library preparation	/5Phos/rArUrArUrArGrG rNrNrNrNrN rArGrArUrCrGrGrArArGrArGrCrGrUrCrGrUrGrUrArG/3SpC3/
RNA_X1B	Library preparation	/5Phos/rArArUrArGrCrA rNrNrNrNrN rArGrArUrCrGrGrArArGrArGrCrGrUrCrGrUrGrUrArG/3SpC3/
RNA_X2A	Library preparation	/5Phos/rArArGrUrArUrA rNrNrNrNrN rArGrArUrCrGrGrArArGrArGrCrGrUrCrGrUrGrUrArG/3SpC3/
RNA_X2B	Library preparation	/5Phos/rArGrArArGrArU rNrNrNrNrN rArGrArUrCrGrGrArArGrArGrCrGrUrCrGrUrGrUrArG/3SpC3/
RiL19	Library preparation	/5Phos/AGAUCGGAAGAGCGUCGUG/3SpC3/
rand103Tr3	Library preparation	/5Phos/NNNNNNNNNNAGATCGGAAGAGCACACGTCTG/3SpC3/
AR17	Library preparation	ACACGACGCTCTTCCGA
PCR_F_D501 NextSeq	Library amplification	AATGATACGGCGACCACCGAGATCTACAC**AGGCTATA**ACACTCTTTCCCTACACGACGCTCTTCCGATCT
PCR_F_D502 NextSeq	Library amplification	AATGATACGGCGACCACCGAGATCTACAC**GCCTCTAT**ACACTCTTTCCCTACACGACGCTCTTCCGATCT
PCR_F_D503 NextSeq	Library amplification	AATGATACGGCGACCACCGAGATCTACAC**AGGATAGG**ACACTCTTTCCCTACACGACGCTCTTCCGATCT
PCR_F_D504 NextSeq	Library amplification	AATGATACGGCGACCACCGAGATCTACAC**TCAGAGCC**ACACTCTTTCCCTACACGACGCTCTTCCGATCT
PCR_R_D701	Library amplification	CAAGCAGAAGACGGCATACGAGAT**CGAGTAAT**GTGACTGGAGTTCAGACGTGTGCTCTTCCGATC
PCR_R_D702	Library amplification	CAAGCAGAAGACGGCATACGAGAT**TCTCCGGA**GTGACTGGAGTTCAGACGTGTGCTCTTCCGATC
PCR_R_D703	Library amplification	CAAGCAGAAGACGGCATACGAGAT**AATGAGCG**GTGACTGGAGTTCAGACGTGTGCTCTTCCGATC
PCR_R_D704	Library amplification	CAAGCAGAAGACGGCATACGAGAT**GGAATCTC**GTGACTGGAGTTCAGACGTGTGCTCTTCCGATC
